# Combined Trapped Ion Mobility and Infrared Ion Spectroscopy Study of Protonation Sites in Aromatic Amines

**DOI:** 10.1021/jasms.5c00164

**Published:** 2025-08-21

**Authors:** Laura Finazzi, Lara van Tetering, Jelle L. Schuurman, Jonathan Martens, Giel Berden, Jos Oomens

**Affiliations:** † FELIX Laboratory, Institute for Molecules and Materials, 530588 Radboud University Nijmegen, Toernooiveld 7, 6525ED Nijmegen, The Netherlands; ‡ van’t Hoff Institute for Molecular Sciences, 6029 University of Amsterdam, Science Park 904, 1098XH Amsterdam, The Netherlands

**Keywords:** trapped ion mobility, infrared ion spectroscopy, protonation sites, aminonaphthalene, aminoanthracene

## Abstract

The protonation site of aromatic amines in the gas phase has been under substantial debate, as it involves a subtle competition between the higher electronegativity of the amine nitrogen and the better charge delocalization ability of the fused aromatic rings. Previous studies have unambiguously shown, especially by ion mobility measurements, that higher-energy tautomers are easily observed depending on the experimental conditions in the ion source, including voltage settings and the type of solvent used in spray sources. Here, we use a combination of ion mobility and ion spectroscopy and focus on the tautomeric structure *after* ion mobility separation, in particular for protonated 1-aminonaphthalene and 1-aminoanthracene. We employ an atmospheric pressure chemical ionization (APCI) source, with a direct insertion probe to avoid any solvent influence, mounted on an FTICR mass spectrometer with a trapped ion mobility (TIMS) unit and optical access to the ions to perform infrared (IR) multiple-photon dissociation spectroscopy using the Free-Electron Laser for Infrared eXperiments (FELIX). TIMS analysis indeed reveals the presence of both N- and C-protonated species, but the IR spectra recorded in the ICR cell also suggest that mobilization and scrambling of the proton occur after TIMS separation. We computationally investigate the energetics of tautomerization and experimentally explore ion activation after TIMS separation.

## Graphical abstract

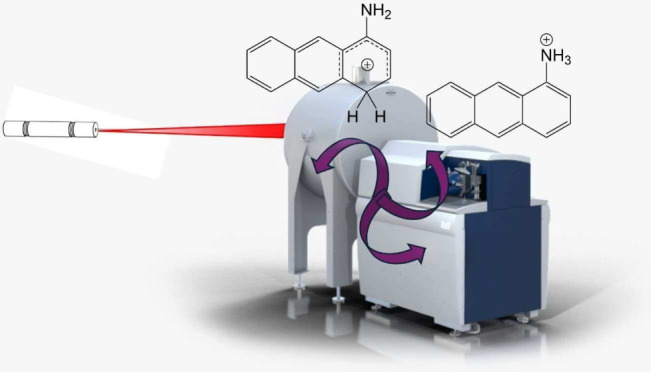

## Introduction

The preferential protonation site of aniline and its derivatives has been a subject of much debate and extensive study.^1–16^ In solution, the amine nitrogen is the most basic site in aniline, so protonation leads to the formation of an ammonium species (−NH_3_^+^). In the gas phase of a mass spectrometer, however, the actual protonation site of aniline is often ambiguous. Theoretical studies at the AM1 and density functional theory (DFT) levels of theory show that particularly the carbon atom located *para* to the amine moiety forms an energetically competitive protonation site, about 8 kJ mol^–1^ lower in energy than protonation on the amine nitrogen.^12,17^ However, earlier theoretical studies using different levels of theory had proposed alternative energetic orderings, fueling the debate concerning the gas-phase protonation site.^1,8,10,18–20^ Experimental studies suggest that several instrumental factors, such as ionization conditions, can yield either N- or C-protonated tautomers.^21–23^ The N-protonated isomer of aniline can be kinetically stabilized in the gas phase^6,12,24,25^ depending on the experimental conditions, especially on whether ions are produced through gas-phase chemical ionization or through electrospray ionization, and in the latter case, on the solvent proticity and polarity.^3,5,7,26^

Intrinsically, protonation on the amine or on one of the aromatic carbon atoms involves the competition between the higher electronegativity of the nitrogen atom and the better charge delocalization ability of the aromatic moiety. As the size of the aromatic system increases upon going from aniline to larger polycyclic aromatic amines, the efficacy of charge delocalization gradually improves, increasing the stability of ring-protonated tautomers relative to that of the amine-protonated tautomer. Figure 1 displays this trend, presenting computed proton affinities of the amine nitrogen and the C atom located *para* to the amine moiety on the same ring, which gives the lowest-energy C-protonated tautomer in all cases. Note the close proximity of proton affinities for aniline and the observation that DFT and MP2 results show similar trends but with significant discrepancy in absolute value for the proton affinity of the amine, reversing the relative stabilities for aniline. Overall, these results align with computed proton affinities reported for 1-aminopyrene^27^ and 1- and 2-aminonaphthalene.^28^

**Figure 1:**
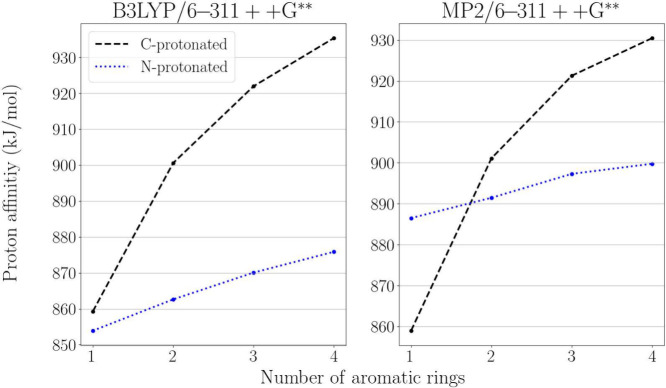
Proton affinity of the amino group (blue) and the *para*-carbon atom (black) in amino acenes as a function of the number of aromatic rings, computed at B3LYP/6-311++G** (left panel) and MP2/6-311++G** (right panel) levels of theory.

In addition to these intrinsic molecular properties, various studies have shown that experimental factors can influence the actual protonation process, potentially favoring the amino group as the kinetically stabilized protonation site. To establish the protonation site experimentally, UV ion spectroscopy and ion mobility spectrometry (IMS) were mainly employed. Jouvet and Noble reported that aminopyrene preferably protonates on one of the skeletal carbon atoms based on absorption in the visible region, whereas the amino protonated tautomer displays absorption in the UV.^28^ Kumar and Attygalle recently employed traveling wave IMS to explore how conditions in the ion source can be adjusted to obtain different tautomeric structures of protonated 1- and 2-aminonaphthalene.^29^ The influence of ionization conditions on tautomer formation was also investigated for aminobenzoic and aminophthalic acids, where isomer abundances were probed by drift-tube IMS.^30^

The facile tautomerization occurring in the ion source as revealed in these studies^29–31^ also raises questions on the stability of structures under conditions of IMS. IMS separates and analyzes gaseous ions based on their mobility through a buffer gas,^32^ and the question whether and to what extent an IMS measurement could alter the structure of an analyte ion has often been addressed.^33–35^ Ion heating can occur during or after mobility separation, affecting collision cross section measurements and possibly inducing conformational changes or even isomerization or dissociation.^36–39^ Thermometer ions have been used to quantify ion heating during IMS,^40–43^ providing insights into internal energy and effective temperature of the ions as they traverse the IMS mass spectrometer.

Conventional IMS instruments can detect structural changes induced in the ionization source prior to IMS analysis. However, post-IMS ion structures can only be probed by tandem-MS based methods,^44–46^ which inherently limits the ability to directly characterize subtle structural differences. More sophisticated analysis of structural modifications arising post-IMS, e.g., through spectroscopy or a second IMS stage, requires mostly dedicated, nonconventional instrumentation.^47–58^

In this study, we combine ion mobility with infrared ion spectroscopy (IRIS) to probe the tautomeric structure of 1-aminonaphthalene and 1-aminoanthracene after separation by ion mobility. In particular, we employ trapped ion mobility spectrometry (TIMS) and IR multiple-photon dissociation (IRMPD) spectroscopy to analyze the site of protonation and determine whether structural changes may occur post-TIMS.

## Methods

### Mass Spectrometry

Experiments were performed on a modified FTICR MS instrument (SolariX XR, Bruker Daltonics, Bremen, Germany) equipped with a 7-T superconducting magnet (Maxwell magnet, Bruker BioSpin, Wissembourg, France). The instrument is equipped with a trapped ion mobility spectrometry (TIMS) stage^59^ and is coupled to the beamline of the Free-Electron Laser for Infrared eXperiments (FELIX),^60^ as schematically shown in Figure 2. This setup was installed recently and allows us to record IR spectra of TIMS-separated and *m*/*z*-selected ions.^61^

**Figure 2:**
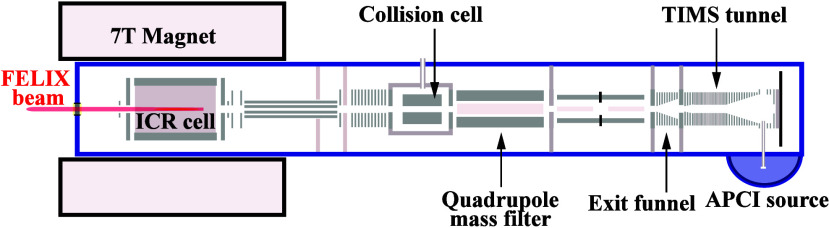
Layout of the FTICR-MS (Bruker SolariX XR) showing the locations of the TIMS unit, the collision cell, and IR laser beam used for IRMPD spectroscopy in the ICR cell.

1-Aminonaphthalene (99%) and 1-aminoanthracene (99%) were purchased from Sigma-Aldrich (St. Louis, USA) and used without further purification. Ions were generated with an atmospheric pressure chemical ionization (APCI) source equipped with a direct insertion probe (DIP).^62^ The solid samples were deposited in small quantities on the glass capillary and inserted into the DIP assembly, where the molecules sublimed and reached the corona discharge. This method resembles that used by Kumar and Attygalle^29^ and invokes protonation in the gas phase, avoiding any possible influence of solvent media on the site of protonation. Ions then travel through a glass capillary into the source vacuum housing. The conventional entrance funnel is replaced by a TIMS tunnel with ion funnels at the entrance and exit (TIMS 0.5, Bruker, Billerica, MA).^59^ This device can be used to mobility select the incoming ions, or it can be used as a standard ion funnel transferring the ions to an RF octopole ion guide, followed by a quadrupole mass filter. A National Instruments LabView program controls the TIMS settings together with an adapted pulse program that synchronizes its operation to the FTICR MS data acquisition sequence. When the TIMS unit is employed as a mobility filter, ions of a selected mobility accumulate in the collision cell, where ions can also be subjected to collision-induced dissociation (CID). After passing through a hexapole guide, ions are transferred to a dynamically harmonized ICR cell (ParaCell, Bruker Daltonics, Bremen, Germany),^63^ where the *m*/*z*-analysis takes place and where IRMPD spectroscopy can be performed with the FELIX beam reaching the ions through a ZnSe window mounted on the back UHV flange of the vacuum system.^61^

### IRMPD Spectroscopy

In the current experiments, ions were irradiated with 10 macropulses of FELIX; each macropulse is ∼10 μs long and has an energy of 20–130 mJ depending on the emission wavelength. Macropulses are emitted at a repetition rate of 10 Hz and with a bandwidth of 0.5% of the center frequency. IR spectra are recorded for mass (and mobility) selected features by acquiring a series of mass spectra as the laser frequency is tuned across the spectroscopic range of interest. Whenever the laser frequency is resonant with a vibrational transition of the selected ion, the absorption of multiple (10–100) photons occurs and induces unimolecular dissociation, as detected in the mass spectrum. An IRMPD spectrum is reconstructed from the individual mass spectra by relating the precursor ion intensity (*P*) to the total fragment ion intensity as a function of laser wavelength

1 $$\mathrm{Yield}=\frac{\underset{i}{\sum }f_{i}}{P+\underset{i}{\sum }f_{i}}$$

where *f*_*i*_ is the intensity of each fragment ion *i* produced by the IR irradiation. The IRMPD yield ranges between 0 and 1. The fragment fluence is a better proxy for the absorbance and is derived as

2 fragmentfluence=−ln(1−Yield)

A linear correction is applied to the fragment fluence to account for wavelength dependent variations in laser power and for the number of laser pulses employed.^64^ Wavelength calibration is achieved with a grating spectrometer.

### Trapped Ion Mobility Spectrometry

Ion mobility spectrometry separates and characterizes ions based on their mobility through a buffer gas, a property closely related to their 3-dimensional shape.^65^ Trapped ion mobility spectrometry (TIMS) constitutes the reversal of the classical drift cell analyzer:^59,66–68^ instead of ions moving through a stationary gas as in a drift cell, TIMS is based on holding the ions stationary in a moving column of gas exerting a forward drag force while being slowed by an opposing electric field.^66^ An electric field gradient induces spatial separation of the ions along the tunnel axis that is dependent on their mobility, *K*, as ions are immobilized at the point where the forward drag force and the backward electric force cancel. By scanning the voltage *V*_hold_ that sets up the field gradient (and thus the electric force), ions of different collisional cross sections (CCSs) elute at different times so that a mobilogram is recorded.^69^ The instantaneous hold voltage at which ions of specific mobility elute (*V*_elute_) is empirically related to the mobility *K* as

3 $$K=A+\frac{B}{V_{elute}-V_{out}}$$

where *A* and *B* are empirical parameters that are fitted using a linear calibration with an Agilent low-concentration tune mix.^70^ In this study, TIMS provides tautomer separation and aids to identify ions based on their CCSs. TIMS separations performed in this work used N_2_ as a buffer gas at approximately 300 K and 1–2 mbar. Parameters for TIMS operation were optimized for maximum ion signal (in view of the ion spectroscopy to be performed subsequently) and are reported in the Supporting Information; these generic settings can be regarded as harsh.^43^

### Computational Methods

Theoretical IR spectra were generated for the possible protonation tautomers of 1-aminonaphthalene and 1-aminoanthracene using density functional theory (DFT) at the B3LYP/6-31++G­(d,p) and B3LYP-D3­(BJ)/6-31+G­(d,p)^71^ levels of theory employing the Gaussian 16 software package^72^ as installed at the Snellius supercomputer at SURFsara, Amsterdam. Furthermore, single-point MP2/6-31++G­(d,p) calculations were performed to verify the relative energies of the different structures. Geometry optimizations were performed with standard convergence criteria, and vibrational spectra were computed within the harmonic oscillator approximation. To account for anharmonicity, harmonic frequencies were scaled by a factor of 0.975 within the fingerprint region (2000–600 cm^–1^).^73^ A revised scaling factor of 0.946 was applied to frequencies calculated for the C–H and N–H stretches; this factor brings the strongest N–H stretch band of mobility peak B of protonated 1-AA exactly in overlap with the calculated band for the C3-protomer (see below).

Transition state (TS) calculations were performed to elucidate the energetics of proton migration along the aromatic ring. TS geometry optimization and frequency calculations were performed following quasi-Newton synchronous transit (QST3) calculations. The stationary points identified along the reaction pathways were verified to be either first-order transition states or local minima by confirming the presence of one or zero imaginary frequency, respectively. Visualization of the corresponding normal mode verified that the TS structures are indeed saddle points connecting the reactant and the product. In addition, intrinsic reaction coordinate (IRC) calculations were performed to verify that the TSs connect the intended reactant and the product. Gibbs energies were used to calculate the activation energy barriers.

## Results and Discussion

Table 1 presents computed gas-phase site-specific proton affinities for 1-aminonaphthalene (1-AN) and 1-aminoanthracene (1-AA) at different levels of theory, enabling one to evaluate the most likely tautomeric structures formed upon protonation. Tautomers protonated at one of the carbon atoms are denoted as 1-ANC_*n*_H^+^ or 1-AAC_*n*_H^+^, with *n* indicating the C atom where protonation occurs (see Figure 3 for atom numbering). For both species, carbon atom C3 is located *para* to the amine and has the highest proton affinity of all C atoms; it is also higher than that of the amino N atom. Furthermore, the energy gap between N- and C-protonated tautomers increases with the size of the aromatic system, as can also be seen in Figure 1. The MP2/6-31++G­(d,p) values deviate from the B3LYP/6-31++G­(d,p) values: at the MP2-level, the N-protonated tautomer is more stable than what is predicted at the B3LYP level. To investigate if this discrepancy is due to B3LYP’s lack of dispersion, we employed the D3­(BJ) dispersion correction, which gives an energy ordering identical with that for uncorrected B3LYP. Additionally, geometry optimization at higher levels of theory (B3LYP-D3­(BJ)/aug-cc-pVTZ) were performed for the lowest-energy isomers of 1-AN, giving again an energy ordering identical with that of B3LYP/6-31++G­(d,p). Single-point calculations at the CCSDT level also predict the C_para_-protonated tautomer to be the most stable isomer, although the amino-protonated tautomer is now lower than the C1-protonated tautomer. Although the origin of the deviation between B3LYP and MP2 remains unclear, we conclude that it is likely not due to a different treatment of dispersion.

**Figure 3:**
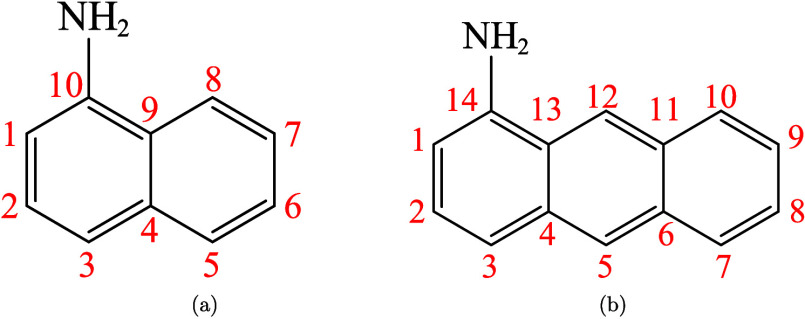
Molecular structures of (a) 1-aminonaphthalene and (b) 1-aminoanthracene with carbon atom labeling.

**Table 1:** Relative Gibbs Energies (at 298 K) in kJ mol^–1^ of Protonated 1-AN and 1-AA, Calculated at Different Levels of Theory

1-ANH^+^			
Level Basis Set	B3LYP 6-31++G(d,p)	MP2 6-31++G(d,p)	B3LYP-D3(BJ) 6-31++G(d,p)
1-ANC_3_H^+^	0	0	0
1-ANC_1_H^+^	16.0	18.6	15.9
1-ANNH_3_^+^	37.9	11.9	35.3
1-ANC_5_H^+^	62.2	84.5	61.6
1-ANC_7_H^+^	66.9	89.7	66.9
1-ANC_8_H^+^	83.2	92.1	83.8
1-ANC_6_H^+^	98.5	108.6	99.5
1-ANC_2_H^+^	99.9	116.3	100.5
1-ANC_9_H^+^	116.6	124.3	114.2
1-ANC_10_H^+^	137.1	139.1	137.7
1-ANC_4_H^+^	163.4	164.1	161.5

1-AAH^+^			
Level Basis Set	B3LYP 6-31++G(d,p)	MP2 6-31++G(d,p)	B3LYP-D3(BJ) 6-31++G(d,p)
1-AAC_3_H^+^	0	0	0
1-AAC_1_H^+^	12.7	12.7	11.3
1-AAC_5_H^+^	18.1	29.3	20.4
1-AAC_12_H^+^	38.1	37.3	38.8
1-AANH_3_^+^	51.9	23.9	48.9
1-AAC_7_H^+^	61.5	86.0	65.7
1-AAC_9_H^+^	72.6	98.5	71.2
1-AAC_10_H^+^	81.8	90.5	82.8
1-AAC_8_H^+^	93.8	103.8	94.3
1-AAC_2_H^+^	94.5	109.9	94.9
1-AAC_14_H^+^	122.2	123.2	122.7
1-AAC_13_H^+^	133.9	142.1	131.6
1-AAC_11_H^+^	149.7	168.1	147.7
1-AAC_4_H^+^	164.4	169.5	162.3
1-AAC_6_H^+^	168.7	168.4	166.5

### Trapped Ion Mobility Spectrometry

Given the ambiguity of the protonation site reported in the literature and predicted by theoretical calculations by us and others, TIMS mobilograms of the [M + H]^+^ ions of 1-AN and 1-AA produced with the APCI source were recorded; see Figure 4. The DIP-APCI ion source does not involve any solvent; the sample sublimes directly from the solid to the gas phase, where protonation occurs during the corona discharge. The mobilograms reveal the presence of two and three distinct isomeric species for 1-AN and 1-AA, respectively, presumably corresponding to different protonation isomers (referred to as protomers in the following). According to CCS calculations and the IMS profiles previously reported,^29,31^ N-protomers have higher CCS values compared to C-protomers as the delocalized charge in C-protomers, indicated for instance by their smaller dipole moment, leads to weaker charge-induced dipole interactions with the N_2_ buffer gas.^48,74^ Therefore, for both 1-AN and 1-AA, the peak eluting at the lowest TIMS hold voltage can presumably be assigned as the N-protomer, followed by a somewhat broader peak attributed to two or more C-protomers, whose CCS values are close and therefore cannot be separated further on our TIMS. For 1-AA, the relative integrated peak intensity associated with the C-protomers is higher than that for 1-AN, which is rationalized by the greater thermodynamic preference for C-protonation (Table 1).

**Figure 4:**
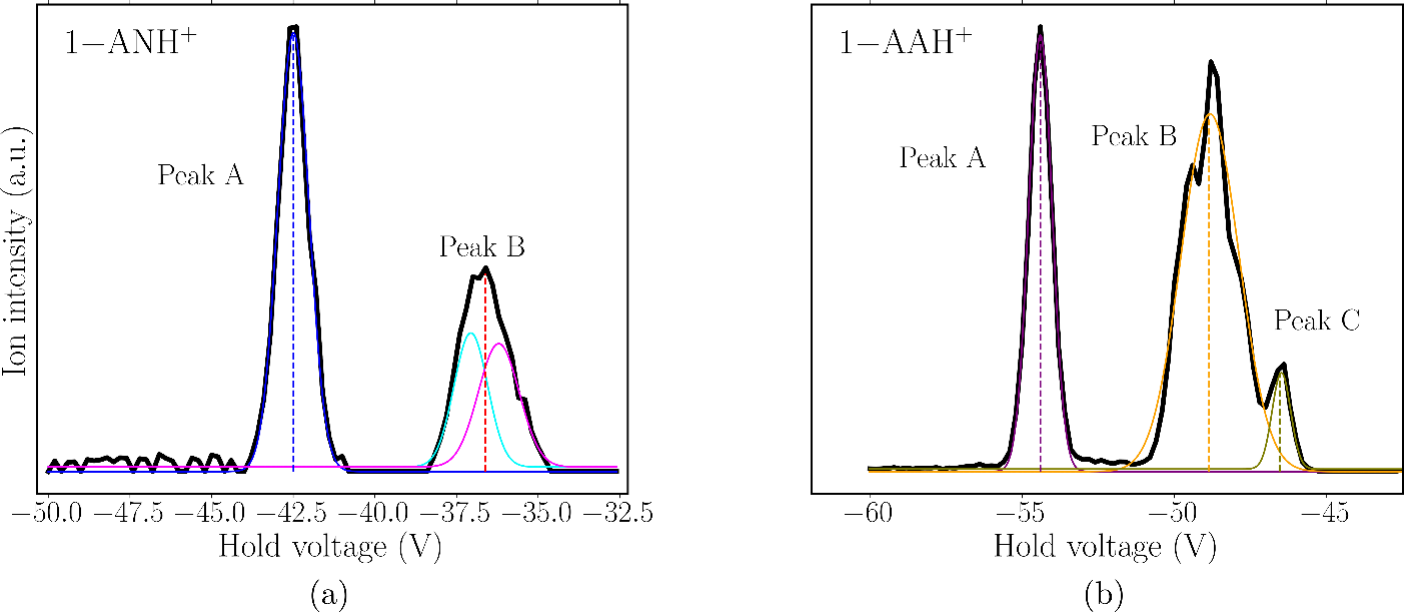
TIMS mobilograms of mass-selected (a) 1-ANH^+^ and (b) 1-AAH^+^. The black, thick lines correspond to the recorded mobilograms. The colored thin lines are Gaussian fits of the experimental peaks. The dashed vertical lines correspond to the *V*_elute_ values employed to select specific tautomers for the IRMPD spectroscopy experiments.

Hence, in line with refs 29 and 31 , our experiments show that the protomers can be analyzed by ion mobility, but the question remains whether the selected isomer is retained until probed by IRMPD spectroscopy in the ICR cell. Ion mobility (in whatever form) relies on interaction with a buffer gas, which can possibly promote structural changes or even dissociation.^33,34,38,40–44^ The tautomerization reactions connecting the different protomers of the amino-acenes involve barriers of intermediate energy, typically smaller than dissociation reactions but larger than conformational changes. Therefore, we spectroscopically characterize the ion structure after mobility separation to investigate whether such an isomerization occurs.

### IRMPD Spectroscopy on Mobility-Selected Ions

To characterize the protonation site of 1-ANH^+^ and 1-AAH^+^ beyond theoretical calculations and TIMS data, IR spectra of the *m*/*z* features of interest are recorded with and without TIMS selection prior to IR interrogation. With the TIMS switched off, ions of all cross sections are transmitted to the ICR cell where their IRMPD spectrum is recorded; see Figure S1 in the SI. The mobility-selected IR ion spectra are obtained by fixing *V*_elute_ at a constant value indicated by the dashed lines in Figure 4, while stepping up the FELIX laser wavelength. For 1-AAH^+^, IRMPD spectra for TIMS-separated ions of peaks A, B, and C are shown in Figure 5b. For 1-ANH^+^ in Figure 5a, the C-protonated tautomers are not resolved in our TIMS (Figure 4a), so we recorded an IRMPD spectrum with *V*_elute_ at the midpoint of the broadened mobility peak, presumably corresponding to a mixture of at least two C-protomers.

**Figure 5:**
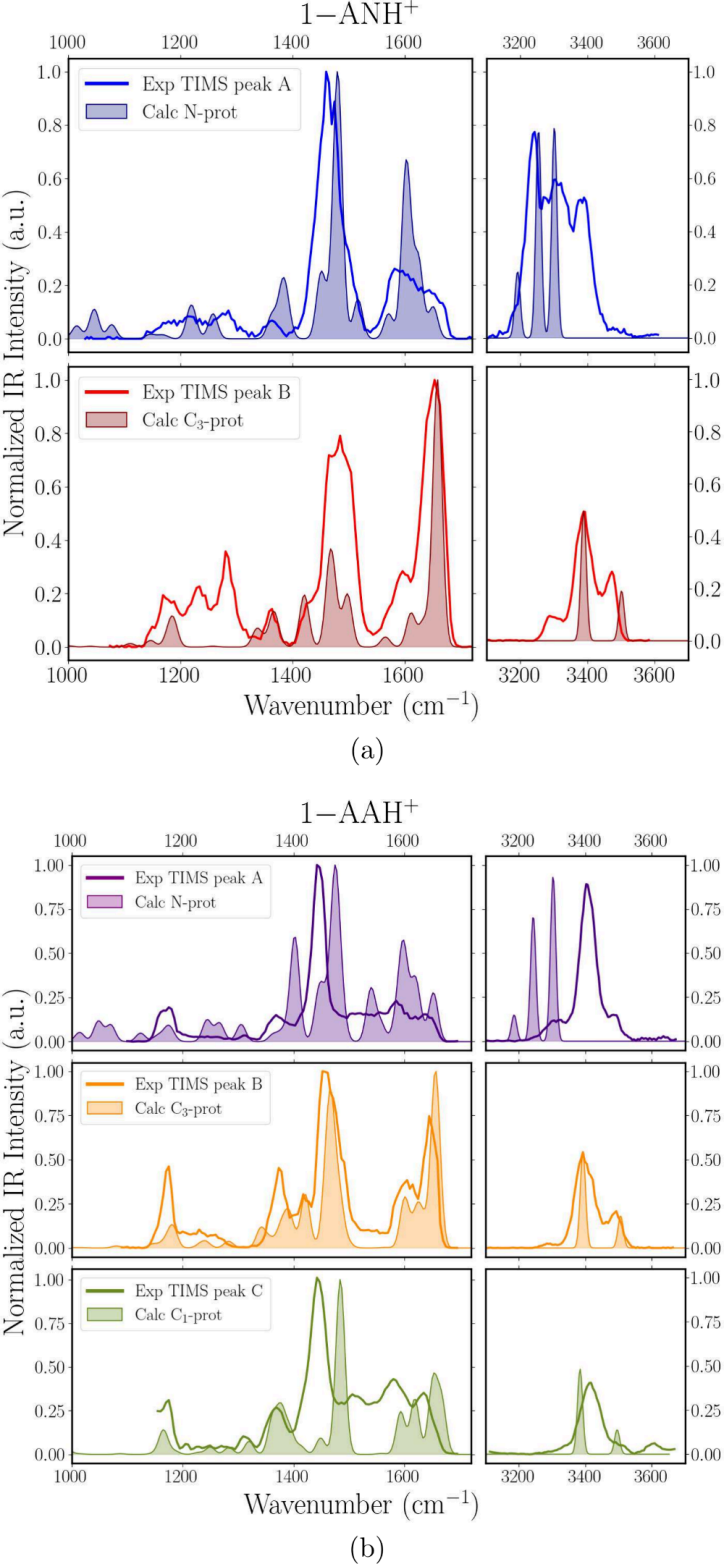
Experimental IRMPD spectra of TIMS-selected (a) 1-ANH^+^ and (b) 1-AAH^+^ ions (thick lines), compared to theoretical spectra of selected protomers (shaded plots): N- and C3-protonated for 1-ANH^+^ and N-, C3-, and C1-protonated for 1-AAH^+^ (top to bottom).

Before presenting a full analysis of the IR spectra based on DFT-predicted IR spectra for the different protomers, it is useful to qualitatively inspect the spectra in the 3 μm region (Figure 5), where one expects to observe clear differences between N-protonated −NH_3_^+^ species, having three NH-stretch modes, and C-protonated species (−NH_2_), having two NH-stretches, as clearly displayed by the distinct computed spectra in Figure 5. For 1-ANH^+^, the two experimental mobility-selected spectra indeed have a distinct appearance, although the bands partially overlap. The IR bands of TIMS peak A corresponding to the N-protonated isomer appear more red-shifted compared with those of peak B, in agreement with the weaker N–H bonds in an ammonium moiety versus an amino moiety. In contrast to 1-ANH^+^, the mobility-separated ions for 1-AAH^+^ feature very similar spectral profiles in the N–H stretch range with just slight relative intensity differences. This common spectral signature resembles that computed for C-protonated species.

This qualitative spectral analysis leads to the preliminary conclusion that, for 1-ANH^+^, the mobility-separated species remain largely in their selected tautomeric state until they reach the ICR cell, where IR spectroscopic analysis takes place. In contrast, in mobility-selected 1-AAH^+^, interconversion of the protonation site occurs after TIMS separation so that the ions end up in the lower-energy C-protonated form when they are probed by IRMPD spectroscopy. In other words, N-protonated ions appear to remain kinetically trapped in 1-ANH^+^ but interconvert to C-protonated isomers in 1-AAH^+^. We perform a computational investigation of the underlying potential energy surfaces (PESs) to further address these observations.

### Computed PES for Tautomerization

The PESs for intramolecular proton transfer in 1-ANH^+^ and 1-AAH^+^ are presented in Figure 6. The calculated pathways show that the initial proton transfer from the amino group to the *ipso*-carbon atom is the rate-limiting step for both systems. The energy of this transition state is ∼210 kJ mol^–1^ in 1-ANH^+^ and nearly 10 kJ mol^–1^ lower for 1-AAH^+^. Moreover, the endothermicity forming the *ipso*-C-protonated intermediate is almost 30 kJ mol^–1^ lower for 1-AAH^+^ than that for 1-ANH^+^. From here on, the PESs of the two systems are qualitatively similar, although the relative energies of all barriers and intermediates are 10–20 kJ mol^–1^ lower in 1-AAH^+^ versus 1-ANH^+^, which is rationalized by the greater charge delocalization for C-protonated isomers in larger aromatic systems. The *meta*-protonated intermediate is thermodynamically unfavorable due to the electron-donating character of the amino group. Our computed PES for 1-ANH^+^ shows good agreement with that reported by Kumar and Attygalle,^29^ who investigated kinetic and thermodynamic control of protonation in 1- and 2-aminonaphthalene.

**Figure 6:**
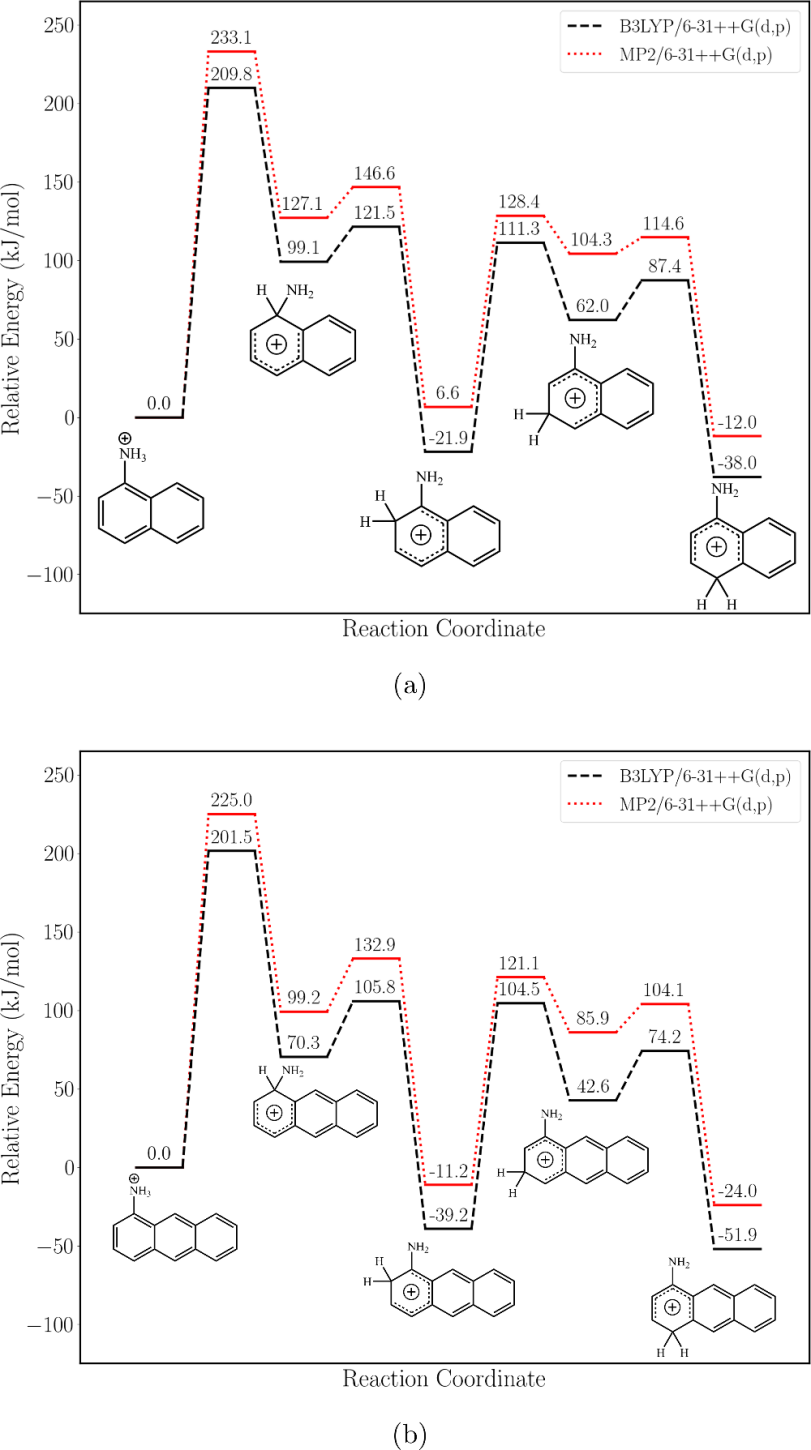
Potential energy surface at the B3LYP/6-31++G­(d,p) (black dashed lines) and MP2/6-31++G­(d,p) (red dotted lines) levels of theory for intramolecular proton hopping in protonated 1-AN (a) and 1-AA (b). Gibbs energies at 298 K in kJ mol^–1^ of transition states and intermediates are relative to those of the N-protomer.

The lower TS-barriers, the lower endothermicity of the intermediates, and the greater the exothermicity of the final *para*-protonated product for 1-AA as compared to 1-AN qualitatively help to explain the observed differences in proton transfer efficiency between the two molecules. N-protonated 1-AA is more likely to undergo intramolecular rearrangement, whereas N-protonated 1-AN is more likely to remain kinetically trapped. Valadbeigi and Causon reported similar behavior occurring in the ion source (prior to ion mobility analysis) for the tautomers of polycyclic aromatic amines.^31^ For 2-aminonaphthalene, they reported a barrier for the first proton migration step of 230 kJ mol^–1^, close to our value of 210 kJ mol^–1^ for 1-AN.

### IR Spectra at Different Collision Energies

The IRIS spectra recorded for the TIMS separated protomers suggest that intramolecular proton transfer around the aromatic moiety occurs after mobility separation, i.e., after the TIMS tunnel and before ions are irradiated in the ICR cell. Recently, Stroganova et al. found evidence for dissociation of multiply charged peptide oligomers occurring at the exit of the TIMS.^39^ Earlier, Morsa et al. noted dissociation of benzylpyridinium “thermometer” ions occurring after the TIMS tunnel and were able to clearly distinguish it from dissociation occurring *before* (likely in the ion source) or *in* the TIMS tunnel.^42^ These authors also argue that dissociation occurring in the TIMS tunnel does not result in a broadening of peaks in the mobilogram; rather, fragment ions are observed at the elution voltage of the fragment, i.e., as if they were created before the TIMS.^42^ Translating to our experiment, the spectroscopic observation of C-protomers while selecting the N-protomer in the TIMS must be due to tautomerization occurring after, and not in, the TIMS tunnel.

The rate-limiting steps in the proton migration pathways of the protonated amino-acenes (Figure 6) are comparable with the dissociation energies of the benzylpyridinium thermometer ions used by Morsa et al. (125 to 225 kJ mol^–1^).^42^ Depending on instrumental settings, a scenario where 1-AAH^+^ undergoes rearrangement but 1-ANH^+^ does not (or less so) is then plausible. The work by Morsa et al. further demonstrated that post-TIMS ion activation occurs especially in the collision cell.^42^ Therefore, we explored the effects of the collision cell voltage on the spectroscopically probed protonation site of 1-AA. We selectively transmit peak A (N-protonation) through the TIMS and manipulate the collision cell voltage to assess spectroscopically to what extent it influences tautomerization to the C-protomer.

The original IRIS spectra in Figure 5 were recorded at a collision cell voltage of −3.0 V, recommended as a generic setting by the manufacturer. Figure 7 compares IRMPD spectra recorded with this voltage set to −5.0 and +1.5 V, with other settings in the −8.0 V to +3.0 V range shown in Figure S2 in the SI. Upon going from −5.0 V (harsh) to +1.5 V (soft), we observe that the IR bands near 3400 and 3500 cm^–1^, correlating with the C-protomer(s), decrease in intensity along with an increase of the band near 3300 cm^–1^, which is due to the N-protomer. Increased formation of the C-protomer at lower collision cell voltages indeed suggests that ion activation and rearrangement occur in the collision cell.

**Figure 7:**
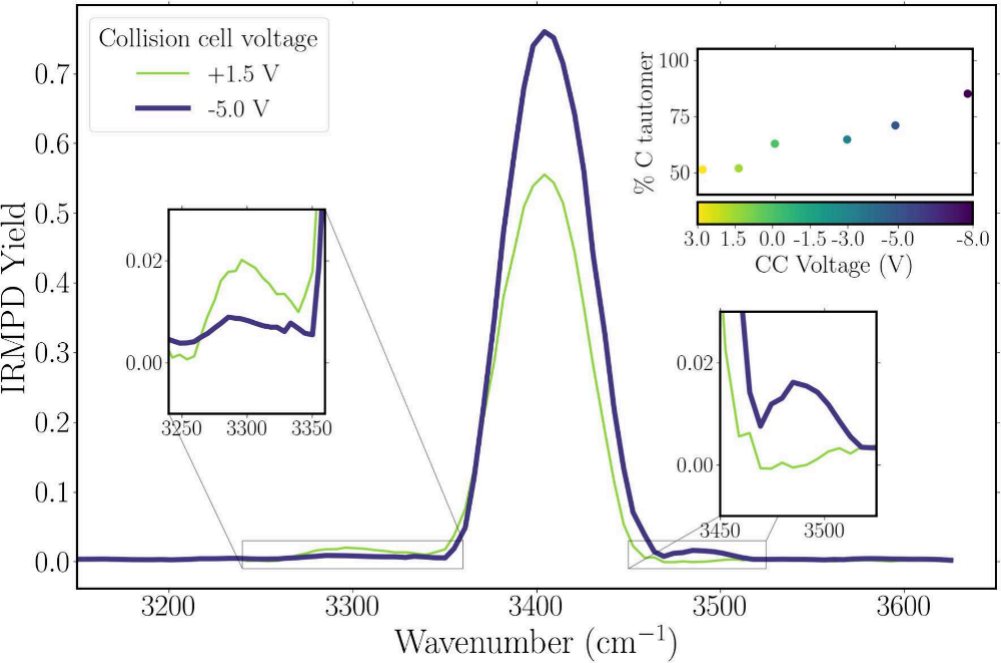
IRMPD spectra of TIMS peak A of 1-AAH^+^ for two different collision cell voltage settings: under harsh conditions (−5.0 V, purple thick line), more tautomerization to the C-protomer is observed than under soft conditions (+1.5 V, green thin line). The inset provides a quantitative view of the C tautomer population as a function of these collision cell settings.

To quantify the extent of tautomerization, we analyzed the IRMPD yield rather than the fragment fluence; see eqs 1 and 2 above and note that the IRMPD yield ranges from 0 to 1.^64^ Furthermore, we removed the lens from the laser beam path to obtain full overlap between laser beam and ion cloud.^61^ At 3400 cm^–1^, we selectively excite the C-protomer (see computed spectra in Figure 5b) and verified that irradiation with 15 laser pulses removes all C-protomers from the ion population (see Figure S3 in the SI). The IRMPD yield of about 0.75 at a voltage of −5.0 V then indicates that 75% of the ions are in the C-protonated form after TIMS selection of the N-protomer. At a voltage of +1.5 V, this fraction drops to about 55%. The fact that, even at the mildest collision cell conditions, a significant fraction of the ions still converts to the C-protomer, likely suggests that ion activation also occurs elsewhere, for instance, in the exit funnel of TIMS, but this requires further investigation. Finally, we note that removal of the focusing lens improves the laser-ion cloud overlap but can suppress weaker bands in the IRMPD spectrum.^64^

### Spectroscopic Analysis

The above analysis suggests that scrambling of the TIMS-separated N- and C-protomers occurs for 1-AAH^+^ but less so for 1-ANH^+^, which aids in the analysis of the IRMPD spectra of the TIMS-selected ions. In the top panel of Figure 5a, the 3 μm spectral range suggests that mobility peak A of 1-ANH^+^ contains mostly the N-protonated species with some admixture of C-protomers suggested by the absorption band at 3385 cm^–1^, coinciding with the strongest band in the bottom panel. In the 1000–2000 cm^–1^ range, the experimental spectrum indeed matches reasonably with the computed spectrum for the N-protomer (1-ANNH_3_^+^). The most intense experimental band at 1450 cm^–1^ corresponds to the umbrella mode of the −NH_3_^+^ group. In the blue, a moderate–intensity, significantly broadened feature is observed, which is due to NH bending, along with nearby weaker absorptions; the shoulder toward ≈1640 cm^–1^ is not well accounted for in the spectrum predicted for the N-protomer and is speculated to be due to the admixture of the C3-protomer, which possesses its strongest band at this frequency, as seen in the bottom panel of Figure 5a.

The 3 μm spectrum of mobility peak B (bottom panel of Figure 5a) suggests a C-protonated ion, and we overlay the experimental spectrum with the predicted spectrum for the lowest-energy C3-protomer. The NH_2_ umbrella mode at 1650 cm^–1^ gives rise to the most intense band in this range of the spectrum, with the shoulder at 1588 cm^–1^ assigned as the CC stretch mode. The strong, broadened band centered at 1475 cm^–1^ is due to closely spaced CC stretch modes, combined with in-plane C–H and N–H bending; the close proximity of two strong bands may artificially enhance the intensity in an IRMPD spectrum.^75,76^ A CH bending mode is present at 1416 cm^–1^ and the CH_2_ scissor mode produces the weak band at 1354 cm^–1^. Between 1200 and 1300 cm^–1^ and at 3300 cm^–1^, more features are observed than those predicted for the C3-protomer. Indeed, the broadened profile of peak B in the mobilogram suggests the presence of multiple C-protomers. Inductive effects shift the NH stretch bands of the *ipso* (C10) and *ortho* (C1) protomers slightly to the red as compared to the other C-protomers (see Figure S4 in the SI), so their presence may explain the absorption near 3300 cm^–1^. Several C-protomers have strong predicted bands near 1300 cm^–1^, providing further evidence for their presence in mobility peak B. The broadened profile of this peak suggests that they are formed in the source, in line with refs 29 and 31 , and post-TIMS activation may lead to further scrambling.

Mobility peak A of 1-AAH^+^ ought to be the N-protomer. However, as argued above, the majority of TIMS-selected ions convert to C-protomer(s) in the collision cell. Indeed, also in the 1000–2000 cm^–1^ range, the computed IR spectrum for the N-protonated ion does not match the experiment (top panel of Figure 5b). The most intense band in the observed spectrum is shifted by 70 cm^–1^ from the predicted umbrella mode in 1-AANH_3_^+^ and, moreover, falls at the same position as that in 1-AAC_3_ *H* ^+^ in the middle panel.

The experimental IR spectrum of TIMS peak B (middle panel in Figure 5b) closely resembles the prediction for the lowest-energy protomer (C3) in both spectral ranges, confirming its assignment to the *para*-carbon protomer. The NH_2_ umbrella mode is located at 1647 cm^–1^ and the bands at 1605 cm^–1^ and 1615 cm^–1^ correspond to CC-stretch modes. The bands at 1455 and 1415 cm^–1^ have mixed CC stretch and NH bending character and the band at 1370 cm^–1^ has mixed CC stretch and CH bending character. The spectrum in the 3 μm range resembles that of C-protonated 1-AN closely, both experimentally and theoretically; even the small apparent shift of the symmetric and antisymmetric NH_2_ stretches relative to theory is reproduced.

TIMS-peak C is a minor feature in the mobilogram of 1-AAH^+^. From its position in the mobilogram and its NH stretch spectrum (bottom panel in Figure 5b), we derive that it must correspond to one or multiple unresolved C-protomers. However, comparison of the experimental spectrum with that predicted for the second lowest-energy C-protomer, 1-AAC_1_H^+^, is disappointing. The strong band at 1440 cm^–1^ is likely associated with the NH_2_ umbrella mode, but its position deviates by about 70 cm^–1^ from the position predicted for the C_1_-protomer. Predicted IR spectra for all C-protomers in addition to C1 and C3 are displayed in Figure S4 in the SI and show significant variation. However, none of them provide a decent match with the experimental spectrum. We note that the main peak, supposedly due to the NH_2_ umbrella mode, overlaps exactly with that of the C3-protomer, i.e., mobility peak B in the middle panel. Moreover, experimental IR spectra for mobility peaks A and C are very similar and deviate only slightly from that for peak B, mainly in the band intensities between 1500 and 1700 cm^–1^. We therefore speculate that the protomer(s) giving rise to mobility peak C tautomerize after the TIMS, just as the N-protomer. The small intensity deviations are then due to some admixture of other C-protomers in the ion population, such as 1-AAC_1_H^+^ and 1-AAC_5_H^+^, which are low in energy and display lower band intensities in the 1500–1700 cm^–1^ range.

In the 3 μm region, vibrational bands related to CH stretching have not been observed in the experiments, in agreement with their low predicted oscillator strengths.

## Conclusions

A novel FTICR-MS platform with combined TIMS and IRIS capabilities was applied to unravel the protonation sites of the amino-acenes 1-AN and 1-AA in the gas-phase. The TIMS mobilogram revealed the presence of both N- and C-protonated tautomers despite significant differences in their thermodynamic stability, analogous to what several ion mobility studies had shown recently as well.^29,31^ Where these previous studies focused mainly on the use of ion mobility analysis to evaluate the distribution of protomers evolving from the ion source, our study employs ion spectroscopy to probe the protomer distribution *after* ion mobility selection. We find that proton migration may occur after the TIMS ion mobility stage, removing the protomer selection imposed and driving the species to the lower-energy C-protomers. We attribute this to ion activation occurring downstream from the TIMS as was previously observed in TIMS as well as traveling-wave and differential IMS instruments using “thermometer” ions.^38,41–44^ Their dissociation thresholds are in the range of the rate-limiting transition states for N-to-C proton transfer (about 200 kJ mol^–1^) in the protonated amino-acenes studied here. Tuning the conditions in the collision cell of the instrument indicates that tautomerization occurs here.

Referring to the conclusion of Kumar and Attygalle that a mobilogram may not reflect the actual distribution of amino-acene protomers in the ion source,^29^ we may add that the mobilogram may not even be representative of the distribution of protomers downstream from the IMS stage. In IM-MS investigations, where ion mobility separation is followed by MS­(/MS) analysis, this may remain unnoticed. While this may have no consequences for studies employing (T)­IMS as an analyzer, one should be cautious using it as a filter, even if no dissociation is observed in the MS analysis.

## Supplementary Material



## Notes

Additional experimental details and data derived from TIMS measurements; calculations of the lowest-energy isomers of 1-AA at additional levels of theory; IRMPD spectra with and without TIMS isolation and equation relating individual mobility-selected IRMPD spectra to spectra recorded without TIMS selection; IRMPD spectra of the TIMS selected N-tautomer of 1-AAH^+^ recorded at different collision cell voltage conditions; computed IR spectra of the other possible tautomers for 1-ANH^+^ and 1-AAH^+^; *XYZ*-coordinates of the B3LYP optimized geometries of the tautomers and transition states involved in the proton scrambling (PDF)

## References

[1] Pollack S. K., Devlin J. L., Summerhays K. D., Taft R. W., Hehre W. J. (1977). The site
of protonation in aniline. J. Am. Chem. Soc..

[2] Maquestiau A., Van Haverbeke Y., Mispreuve H., Flammang R., Harris J. A., Howe I., Beynon J. H. (1980). The Gas Phase Structure of Some Protonated
and Ethylated Aromatic Amines. Org. Mass Spectrom..

[3] Lau Y. K., Kebarle P. (1976). Substituent Effects
on the Intrinsic Basicity of Benzene:
Proton Affinities of Substituted Benzenes. J.
Am. Chem. Soc..

[4] Wood K. V., Burinsky D. J., Cameron D., Cooks R. G. (1983). Site of Gas-phase
Cation Attachment. Protonation, Methylation, and Ethylation of Aniline,
Phenol, and Thiophenol. J. Org. Chem..

[5] Karpas Z., Berant Z., Stimac R. M. (1990). An Ion
Mobility Spectrometry/Mass
Spectrometry (IMS/MS) Study of the Site of Protonation in Anilines. Struct. Chem..

[6] Smith R. L., Chyall L. J., Beasley B. J., Kenttamaa H. I. (1995). The Site
of Protonation of Aniline. J. Am. Chem. Soc..

[7] Nold M. J., Wesdemiotis C. (1996). Differentiation
of N-from C-Protonated Aniline by Neutralization-Reionization. J. Mass Spectrom..

[8] Roy R. K., de Proft F., Geerlings P. (1998). Site of Protonation
in Aniline and
Substituted Anilines in the Gas Phase: A Study via the Local Hard
and Soft Acids and Bases Concept. J. Phys. Chem.
A.

[9] Lee S.-W., Cox H., Goddard W. A., Beauchamp J. L. (2000). Chemistry in Nanodroplets: Studies
of Protonation Sites of Substituted Anilines in Water Clusters Using
FT-ICR. J. Am. Chem. Soc..

[10] Russo N., Toscano M., Grand A., Mineva T. (2000). Proton Affinity and
Protonation Sites of Aniline. Energetic Behavior and Density Functional
Reactivity Indices. J. Phys. Chem. A.

[11] Le H. T., Flammang R., Barbieux-Flammang M., Gerbaux P., Nguyen M. T. (2002). Ionized
Aniline and its Distonic Radical Cation Isomers. Int. J. Mass Spectrom..

[12] Flammang R., Dechamps N., Pascal L., Haverbeke Y., Gerbaux P., Nam P.-C., Nguyen M. (2004). Ring Versus Nitrogen
Protonation of Anilines. Lett. Org. Chem..

[13] Pasker F. M., Solcà N., Dopfer O. (2006). Spectroscopic Identification of Carbenium
and Ammonium Isomers of Protonated Aniline (AnH^+^): IR Spectra
of Weakly Bound AnH^+^-L_
*n*
_ Clusters
(L = Ar, N_2_). J. Phys. Chem. A.

[14] Attygalle A. B., Xia H., Pavlov J. (2017). Influence
of Ionization Source Conditions on the Gas-Phase
Protomer Distribution of Anilinium and Related Cations. J. Am. Soc. Mass Spectrom..

[15] Walker S. W. C., Mark A., Verbuyst B., Bogdanov B., Campbell J. L., Hopkins W. S. (2018). Characterizing the
Tautomers of Protonated Aniline
Using Differential Mobility Spectrometry and Mass Spectrometry. J. Phys. Chem. A.

[16] Naylor C. N., Schaefer C., Kirk A. T., Zimmermann S. (2023). The Origin
of Isomerization of Aniline Revealed by High Kinetic Energy Ion Mobility
Spectrometry (HiKE-IMS). Phys. Chem. Chem. Phys..

[17] Dewar M. J., Dieter K. M. (1986). Evaluation of AM1
calculated proton affinities and
deprotonation enthalpies. J. Am. Chem. Soc..

[18] Chyall L. J., Kenttámaa H. I. (1994). The 4-dehydroanilinium
ion: a stable distonic isomer
of ionized aniline. J. Am. Chem. Soc..

[19] Hillebrand C., Klessinger M., Eckert-Maksić M., Maksić Z. B. (1996). Theoretical
model calculations of the proton affinities of aminoalkanes, aniline,
and pyridine. J. Phys. Chem..

[20] Bagno A., Terrier F. (2001). Carbon and nitrogen
basicity of aminothiophenes and
anilines. J. Phys. Chem. A.

[21] Chai Y., Hu N., Pan Y. (2013). Kinetic and
thermodynamic control of protonation in
atmospheric pressure chemical ionization. J.
Am. Soc. Mass Spectrom..

[22] Campbell J. L., Le Blanc J. C. Y., Schneider B. B. (2012). Probing
electrospray ionization dynamics
using differential mobility spectrometry: The curious case of 4-aminobenzoic
acid. Anal. Chem..

[23] Joyce J. R., Richards D. S. (2011). Kinetic control
of protonation in electrospray ionization. J.
Am. Soc. Mass Spectrom..

[24] Lalli P. M., Iglesias B. A., Toma H. E., de Sa G. F., Daroda R. J., Silva Filho J. C., Szulejko J. E., Araki K., Eberlin M. N. (2012). Protomers:
formation, separation and characterization via travelling wave ion
mobility mass spectrometry. J. Mass Spectrom..

[25] Boschmans J., Jacobs S., Williams J. P., Palmer M., Richardson K., Giles K., Lapthorn C., Herrebout W. A., Lemière F., Sobott F. (2016). Combining density functional
theory
(DFT) and collision cross-section (CCS) calculations to analyze the
gas-phase behaviour of small molecules and their protonation site
isomers. Analyst.

[26] Summerhays K. D., Pollack S. K., Taft R. W., Hehre W. J. (1977). Gas-Phase Basicities
of Substituted Anilines. Inferences about the Role of Solvent in Dictating
Site of Protonation. J. Am. Chem. Soc..

[27] Noble J. A., Dedonder-Lardeux C., Mascetti J., Jouvet C. (2017). Electronic Spectroscopy
of Protonated 1-Aminopyrene in a Cold Ion Trap. Chem.—Asian J..

[28] Noble J. A., Broquier M., Gregoire G., Soorkia S., Pino G., Marceca E., Dedonder-Lardeux C., Jouvet C. (2018). Tautomerism and electronic
spectroscopy of protonated 1- and 2-aminonaphthalene. Phys. Chem. Chem. Phys..

[29] Kumar M., Attygalle A. B. (2024). Manipulating Non-Dissociative Transformations of Gaseous
Ion Ensembles Prior to Ion-Mobility Separation. J. Am. Soc. Mass Spectrom..

[30] Valadbeigi Y., Causon T. (2023). Mechanism of formation and ion mobility separation
of protomers and deprotomers of diaminobenzoic acids and aminophthalic
acids. Phys. Chem. Chem. Phys..

[31] Valadbeigi Y., Causon T. (2023). Monitoring intramolecular
proton transfer with ion
mobility-mass spectrometry and in-source ion activation. Chem. Commun..

[32] Mason, E. A. ; McDaniel, E. W. Transport Properties of Ions in Gases; Wiley Online Library, 1988; Vol. 26.

[33] Merenbloom S. I., Flick T. G., Williams E. R. (2012). How hot are your ions in TWAVE ion
mobility spectrometry?. J. Am. Soc. Mass Spectrom..

[34] Balthasart F., Plavec J., Gabelica V. (2013). Ammonium ion
binding to DNA G-quadruplexes:
Do electrospray mass spectra faithfully reflect the solution-phase
species?. J. Am. Soc. Mass Spectrom..

[35] Gabelica V., Marklund E. (2018). Fundamentals of ion
mobility spectrometry. Curr. Opin. Chem. Biol..

[36] Morsa D., Gabelica V., De Pauw E. (2014). Fragmentation
and isomerization due
to field heating in traveling wave ion mobility spectrometry. J. Am. Soc. Mass Spectrom..

[37] Anwar A., Psutka J., Walker S. W., Dieckmann T., Janizewski J. S., Campbell J. L., Hopkins W. S. (2018). Separating
and probing
tautomers of protonated nucleobases using differential mobility spectrometry. Int. J. Mass Spectrom..

[38] Campbell M. T., Glish G. L. (2018). Fragmentation in the ion transfer
optics after differential
ion mobility spectrometry produces multiple artifact monomer peaks. Int. J. Mass Spectrom..

[39] Stroganova I., Willenberg H., Tente T., Depland A. D., Bakels S., Rijs A. M. (2024). Exploring the Aggregation Propensity
of PHF6 Peptide
Segments of the Tau Protein Using Ion Mobility Mass Spectrometry Techniques. Anal. Chem..

[40] Lermyte F., Sobott F. (2017). A broader view on ion heating in
traveling-wave devices
using fragmentation of CsI clusters and extent of Ḣ migration
as molecular thermometers. Analyst.

[41] Ieritano C., Featherstone J., Haack A., Guna M., Campbell J. L., Hopkins W. S. (2020). How Hot
Are Your Ions in Differential Mobility Spectrometry?. J. Am. Soc. Mass Spectrom..

[42] Morsa D., Hanozin E., Eppe G., Quinton L., Gabelica V., De Pauw E. (2020). Effective Temperature
and Structural Rearrangement
in Trapped Ion Mobility Spectrometry. Anal.
Chem..

[43] Bleiholder C., Liu F. C., Chai M. (2020). Comment on Effective Temperature
and Structural Rearrangement in Trapped Ion Mobility Spectrometry. Anal. Chem..

[44] Morsa D., Gabelica V., De Pauw E. (2011). Effective temperature
of ions in
traveling wave ion mobility spectrometry. Anal.
Chem..

[45] Haler J. R., Massonnet P., Chirot F., Kune C., Comby-Zerbino C., Jordens J., Honing M., Mengerink Y., Far J., Dugourd P. (2018). De Pauw, E. Comparison of Different Ion Mobility Setups
Using Poly (Ethylene Oxide) PEO Polymers: Drift Tube, TIMS, and T-Wave. J. Am. Soc. Mass Spectrom..

[46] Oranzi N. R., Kemperman R. H., Wei M. S., Petkovska V. I., Granato S. W., Rochon B., Kaszycki J., Rotta A. L., Fouque K. J. D., Fernandez-Lima F., Yost R. A. (2019). Measuring the integrity
of gas-phase conformers of sodiated 25-hydroxyvitamin d3 by drift
tube, traveling wave, trapped, and high-field asymmetric ion mobility. Anal. Chem..

[47] Papadopoulos G., Svendsen A., Boyarkin O. V., Rizzo T. R. (2011). Spectroscopy
of
mobility-selected biomolecular ions. Faraday
Discuss..

[48] Warnke S., Seo J., Boschmans J., Sobott F., Scrivens J. H., Bleiholder C., Bowers M. T., Gewinner S., Schollkopf W., Pagel K., von Helden G. (2015). Protomers of benzocaine: Solvent
and permittivity dependence. J. Am. Chem. Soc..

[49] Seo J., Warnke S., Gewinner S., Schollkopf W., Bowers M. T., Pagel K., von Helden G. (2016). The impact
of environment and resonance effects on the site of protonation of
aminobenzoic acid derivatives. Phys. Chem. Chem.
Phys..

[50] Kamrath M.
Z., Rizzo T. R. (2018). Combining
Ion Mobility and Cryogenic Spectroscopy for
Structural and Analytical Studies of Biomolecular Ions. Acc. Chem. Res..

[51] Ben
Faleh A., Warnke S., Rizzo T. R. (2019). Combining Ultrahigh-Resolution
Ion-Mobility Spectrometry with Cryogenic Infrared Spectroscopy for
the Analysis of Glycan Mixtures. Anal. Chem..

[52] Warnke S., Ben Faleh A., Scutelnic V., Rizzo T. R. (2019). Separation and Identification
of Glycan Anomers Using Ultrahigh-Resolution Ion-Mobility Spectrometry
and Cryogenic Ion Spectroscopy. J. Am. Soc.
Mass Spectrom..

[53] Warnke S., Ben Faleh A., Pellegrinelli R. P., Yalovenko N., Rizzo T. R. (2019). Combining ultra-high resolution ion
mobility spectrometry
with cryogenic IR spectroscopy for the study of biomolecular ions. Faraday Discuss..

[54] Buntine J. T., Carrascosa E., Bull J. N., Jacovella U., Cotter M. I., Watkins P., Liu C., Scholz M. S., Adamson B. D., Marlton S. J., Bieske E. J. (2022). An ion mobility
mass spectrometer coupled with a cryogenic ion trap for recording
electronic spectra of charged, isomer-selected clusters. Rev. Sci. Instrum..

[55] Pellegrinelli R. P., Yue L., Carrascosa E., Faleh A. B., Warnke S., Bansal P., Rizzo T. R. (2022). A New Strategy Coupling Ion-Mobility-Selective CID
and Cryogenic IR Spectroscopy to Identify Glycan Anomers. J. Am. Soc. Mass Spectrom..

[56] Yatsyna V., Abikhodr A. H., Faleh A. B., Warnke S., Rizzo T. R. (2022). High-Throughput
Multiplexed Infrared Spectroscopy of Ion Mobility-Separated Species
Using Hadamard Transform. Anal. Chem..

[57] Bansal P., Faleh A. B., Warnke S., Rizzo T. R. (2023). Multistage Ion Mobility
Spectrometry Combined with Infrared Spectroscopy for Glycan Analysis. J. Am. Soc. Mass Spectrom..

[58] Limbach M. N., Lindberg E. T., Olivos H. J., van Tetering L., Steren C. A., Martens J., Ngo V. A., Oomens J., Do T. D. (2024). Taming Conformational Heterogeneity
on Ion Racetrack to Unveil Principles
that Drive Membrane Permeation of Cyclosporines. JACS Au.

[59] Ridgeway M. E., Wolff J. J., Silveira J. A., Lin C., Costello C. E., Park M. A. (2016). Gated trapped ion mobility spectrometry coupled to
fourier transform ion cyclotron resonance mass spectrometry. Int. J. Ion Mobility Spectrom..

[60] Oepts D., van der Meer A. F., van Amersfoort P. W. (1995). The Free-Electron-Laser
user facility
FELIX. Infrared Phys. Technol..

[61] Houthuijs K. J., van Tetering L., Schuurman J. L., Wootton C. A., Gebhardt C. R., Ridgeway M. E., Berden G., Martens J., Oomens J. (2024). A trapped
ion mobility enabled Fourier transform ion cyclotron resonance mass
spectrometer for infrared ion spectroscopy at FELIX. Int. J. Mass Spectrom..

[62] Palotás J., Martens J., Berden G., Oomens J. (2021). Laboratory
IR spectroscopy
of protonated hexa-peri-hexabenzocoronene and dicoronylene. J. Mol. Spectrosc..

[63] Nikolaev E. N., Boldin I. A., Jertz R., Baykut G. (2011). Initial experimental
characterization of a new ultra-high resolution FTICR cell with dynamic
harmonization. J. Am. Soc. Mass Spectrom..

[64] Berden G., Derksen M., Houthuijs K. J., Martens J., Oomens J. (2019). An automatic
variable laser attenuator for IRMPD spectroscopy and analysis of power-dependence
in fragmentation spectra. Int. J. Mass Spectrom..

[65] Gabelica V. (2019). Recommendations for
reporting ion mobility Mass Spectrometry measurements. Mass Spectrom. Rev..

[66] Ridgeway M. E., Lubeck M., Jordens J., Mann M., Park M. A. (2018). Trapped
ion mobility spectrometry: A short review. Int.
J. Mass Spectrom..

[67] Fernandez-Lima F., Kaplan D. A., Suetering J., Park M. A. (2011). Gas-phase separation
using a trapped ion mobility spectrometer. Int.
J. Ion Mobility Spectrom..

[68] Fernandez-Lima F. A., Kaplan D. A., Park M. A. (2011). Note: Integration
of trapped ion
mobility spectrometry with mass spectrometry. Rev. Sci. Instrum..

[69] Silveira J. A., Ridgeway M. E., Park M. A. (2014). High Resolution Trapped Ion Mobility
Spectrometery of Peptides. Anal. Chem..

[70] Stow S. M., Causon T. J., Zheng X., Kurulugama R. T., Mairinger T., May J. C., Rennie E. E., Baker E. S., Smith R. D., McLean J. A., Hann S., Fjeldsted J. C. (2017). An Interlaboratory
Evaluation of Drift Tube Ion Mobility-Mass Spectrometry Collision
Cross Section Measurements. Anal. Chem..

[71] Grimme S., Ehrlich S., Goerigk L. (2011). Effect of
the damping function in
dispersion corrected density functional theory. J. Comput. Chem..

[72] Frisch, M. J. ; Gaussian 16, Revision C.01; Gaussian Inc.: Wallingford, CT, 2016.

[73] Bauschlicher C. W., Ricca A. (2010). On the calculation
of the vibrational frequencies of polycyclic aromatic
hydrocarbons. Mol. Phys..

[74] Lapthorn C., Pullen F., Chowdhry B. Z. (2013). Ion mobility
spectrometry-mass spectrometry
(IMS-MS) of small molecules: Separating and assigning structures to
ions. Mass Spectrom. Rev..

[75] Oomens J., Tielens A. G. G. M., Sartakov B. G., von Helden G., Meijer G. (2003). Laboratory Infrared
Spectroscopy of Cationic Polycyclic
Aromatic Hydrocarbon Molecules. Astrophys. J..

[76] Parneix P., Basire M., Calvo F. (2013). Accurate modeling
of infrared multiple
photon dissociation spectra: The dynamical role of anharmonicities. J. Phys. Chem. A.

