# Supplementary material for: Combined Trapped Ion Mobility and Infrared Ion Spectroscopy Study of Protonation Sites in Aromatic Amines
Source: J Am Soc Mass Spectrom. 2025 Aug 21;36(9):1940–9. doi: 10.1021/jasms.5c00164 (PMC12412157; doi:10.1021/jasms.5c00164)
Supplement: Supplementary file 1 [file js5c00164_si_001.pdf]

Supplementary Information

Combined trapped ion mobility and infrared ion spectroscopy study of protonation sites in aromatic amines

Laura Finazzi[1], Lara van Tetering[1], Jelle Schuurman[1], Jonathan Martens[1], Giel Berden[1], and Jos Oomens[1,2,*]

[1]Radboud University, Institute for Molecules and Materials, FELIX laboratory, Toernooiveld 7, 6525ED Nijmegen, The Netherlands
[2]van 't Hoff Institute for Molecular Sciences, University of Amsterdam, Science Park 904, 1098XH Amsterdam, The Netherlands

*corresponding author: jos.oomens@ru.nl

Table S1: Detailed instrumental parameters for TIMS-IRMPD experiments.

Parameter	Value
APCI: gas temperature	220 °C
APCI: drying gas flow rate	3.5 L/min
APCI: Nebulizer pressure	2.5 bar
APCI: vaporizing temperature	380 °C
APCI: corona needle current	6000 nA
APCI: capillary voltage	4500 V
Skimmer 1	30 V
Accumulation time	1 s / 2 s (*)
Number of TIMS frames	20
Profile Length	48.32 ms
TIMS frame	20
Fill/trap/elute/quench sequence	15/1/24/8 ms
Ion Deflector Open	140 V
Ion Deflector Close	40 V
Funnel In Ramp	50 V
TIMS Ramp, Start Voltage	-50 V, -60 V (*)
TIMS Ramp, Voltage Ramp	1 V
Split Lens Open	1 V
Split Lens Close	-60 V
Collision Cell Voltage	-3 V (**)

(*) For 1-aminonaphthalene and 1-aminoanthracene, the first and second valued have been employed during the experiments, respectively.

(**) This value was initially set according to manufacturer guidelines. We then explored the impact of collision cell voltage by varying it between -8.0 V and +3.0 V.

Table S2: Tautomer ratios derived from TIMS experiments

**1-aminonaphthalene**	Peak Area	Total Area	Ratio
Peak A	7142046	1.23038E7	0.58
Peak B	5161734		0.42

**1-aminoanthracene**	Peak Area	Total Area	Ratio
Peak A	5.74E7	1.78826E8	0.32
Peak B	1.11E8		0.62
Peak C	1.01E7		0.06

Table S3: Computed relative Gibbs energies (at 298 K) in kJ/mol of the lowest energy isomers of 1- aminonaphthalene using additional (higher) levels of theory.

Level	RB3LYP	CCSDT[a]	MP2
Basis set	Aug-CC-pVTZ	Aug-CC-pVTZ	Aug-CC-pVTZ
1-ANC$_3$H$^+$	0	0	0
1-ANC$_1$H$^+$	17.0	20.6	23.6
1-ANNH$^+$	34.8	14.5	0.5

[a] Single-point calculation at the optimized MP2-geometry

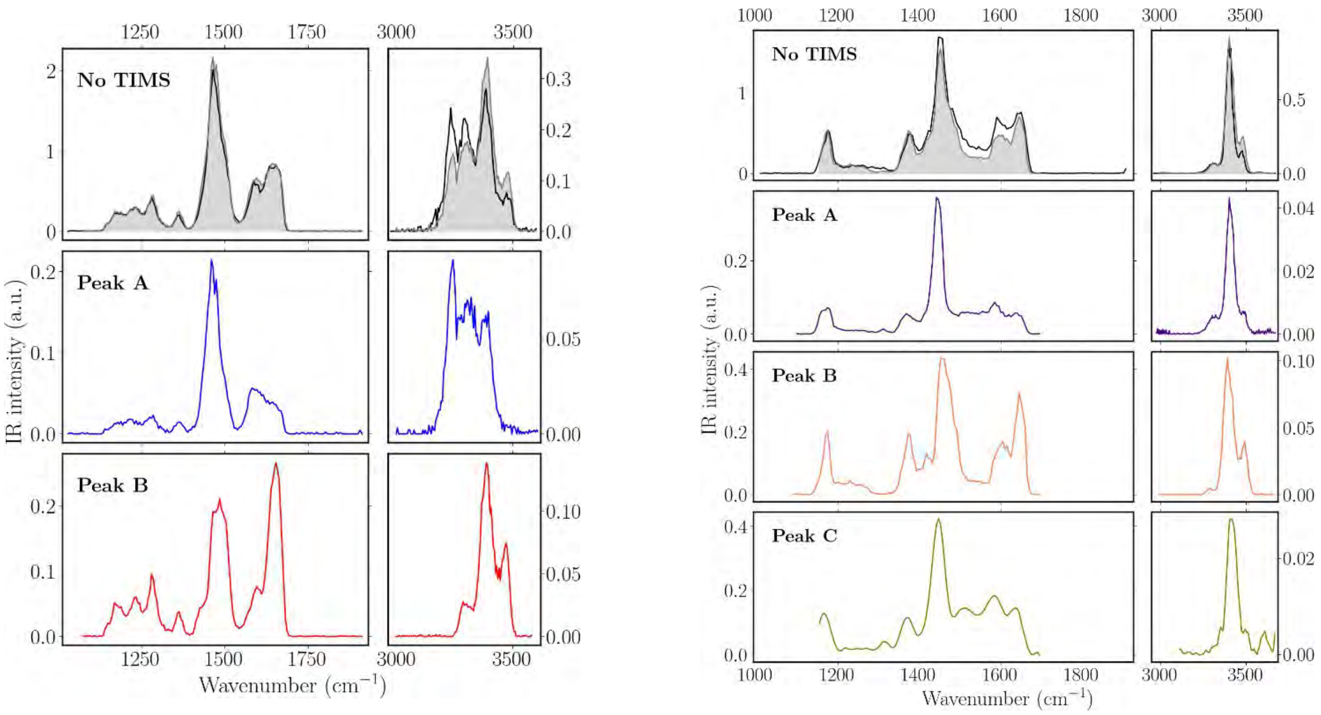

Figure S1: *Experimental IRMPD spectra of 1-ANH$^+$ (left) and 1-AAH$^+$ (right) obtained with TIMS selection off (black lines in top panels) and with mobility selection on, fixing V_{elute} at the values indicated by the vertical lines in the mobilograms in Figure 3 of the main text. The shaded area in the top panels corresponds to the composite IR spectra of all protomers reconstructed from the individual, TIMS-selected IR spectra, using the procedure outlined in the text.*

Note to Figure S1

IRMPD spectra recorded without TIMS selection (black lines in Figure S1) can be related to the individual, mobility-selected IRMPD spectra (colored traces in lower panels), as outlined here. The yield for the non-TIMS selected parent is determined in the conventional way

$$Y_{tot} = \frac{\sum_i f_i}{\sum_i f_i + P} \qquad (1)$$

where P and f_i are the intensities of parent and fragment ions in the "no-TIMS" IRMPD mass spectrum. With TIMS selection on, the yield of each mobility-selected isomer *k* can be determined individually as

$$Y_k = \frac{\sum_i f_{k,i}}{\sum_i f_{k,i} + P_k} \qquad (2)$$

Comparing Eqs. (1) and (2) leads to the conclusion that the yield observed in the no-TIMS IRMPD spectrum is the abundance-weighted average of the isomer-specific yields, in other words,

$$\sum_k x_k Y_k = \sum_k x_k \frac{\sum_i f_{k,i}}{\sum_i f_{k,i} + P_k} \qquad (3)$$

Thus, the righthand part of Eq. (3) shows the breakdown of P and f_i into the individual contributions of each of the isomers *k*, where x_k represents the fractional abundance of each isomer, such that $\Sigma_k x_k = 1$. The numerical values for the fractional abundances can be determined from the integrated intensities of the peaks in the mobilogram, as reported in S3. Using the TIMS-selected IRMPD data and the fractional abundances, all parameters in Eq. (3) are known and can be used to reconstruct a "synthetic" no-TIMS IRMPD spectrum. This composite IRMPD spectrum is presented as the shaded grey spectrum in Figure S1 overlaid onto the experimental no-TIMS IRMPD spectrum. For both 1-ANH$^+$ and 1-AAH$^+$, we find good agreement, also in relative intensities of the IR bands.

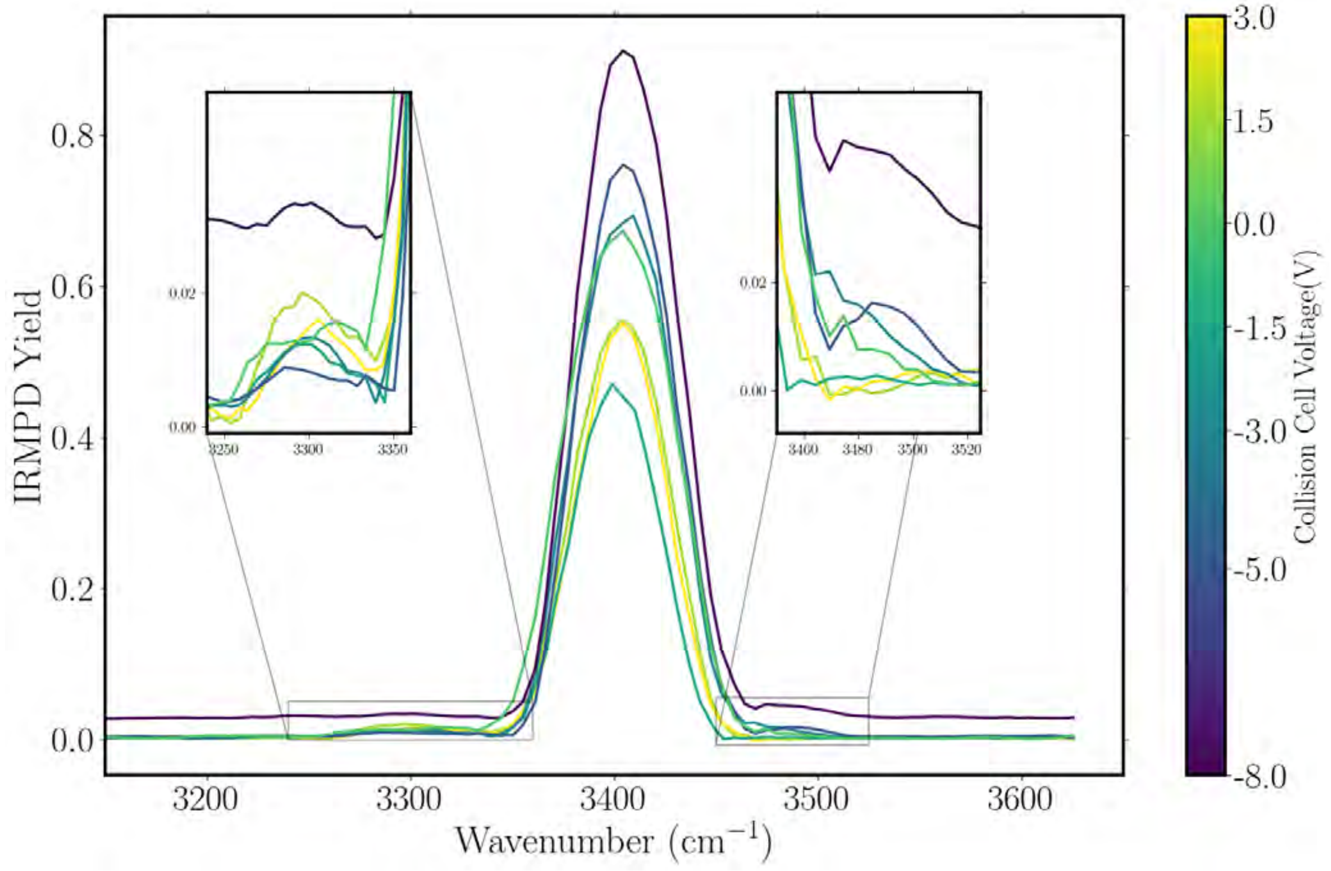

Figure S2: *The IRMPD spectra of 1-AAH$^+$ (TIMS peak A) were recorded under varying collision cell voltages, using always 15 laser pulses so that saturation occurs on the 3400 cm^{-1} band of the C-protomer. A voltage of -8.0 V represents the harshest conditions, while +3.0 V is the mildest setting. The fraction of C-protomers is seen to decrease from about 80% to about 50% as conditions in the collision cell get milder, indicating reduced activation of the TIMS-selected N-protomer. The zoomed inset on the left highlights the band due to the N-protomer at 3300 cm^{-1}, which increases in intensity as the settings get milder. A higher baseline is evident in the -8.0 V spectrum, suggesting that the very harsh collision cell conditions induce some auto-fragmentation. Collision cell voltages outside the -8.0 to +3.0 V range result in severe loss of ion signal. The data was smoothed using a Savitzky-Golay filter with a window length of 10 points and a third-order polynomial, as implemented in the SciPy signal processing library.*

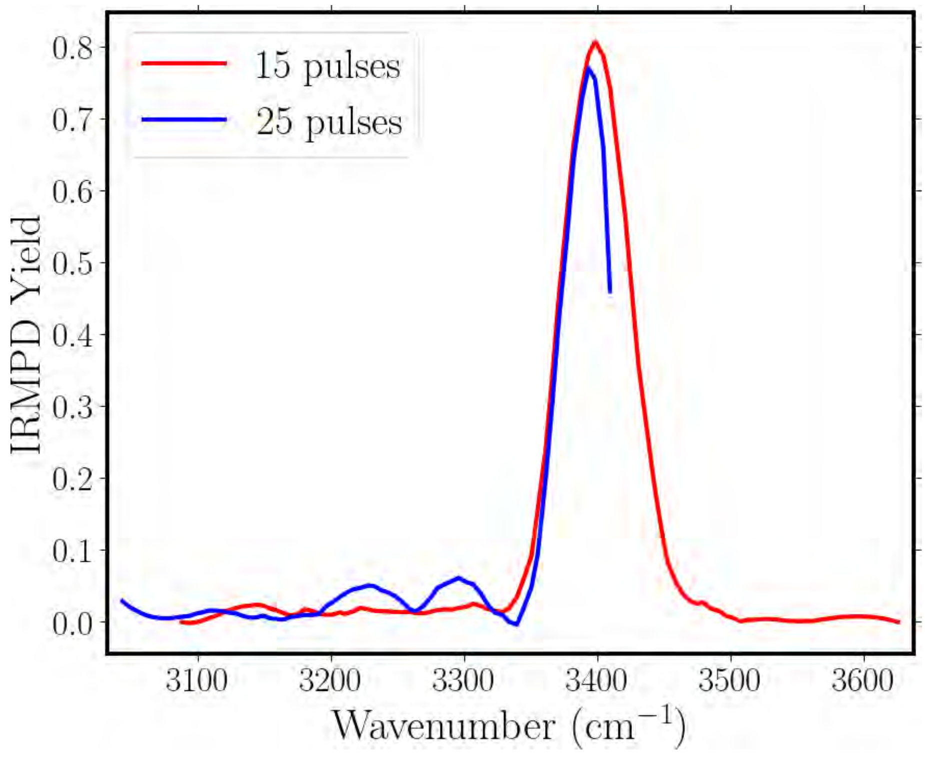

Figure S3: *IRMPD yield spectrum of the TIMS selected N-protomer (TIMS peak A) of 1-AAH$^+$ recorded with 15 and 25 laser pulses. The yield at the 3400 cm^{-1} band, which is due to the C-protomer only, does not change going from 15 to 25 pulses, indicating that all ions that are in the C-protomeric form have dissociated, causing the IRMPD signal to saturate. The saturated IRMPD yield of about 0.8 then reflects the fraction of the ion population in the C-protomeric form.*

Figure S4: *Computed spectra for tautomers of 1-ANC$_n$H$^+$ (on page S7) and of 1-AAC$_n$H$^+$ (on page S8) that are not included in the main body of the paper. Each subplot is identified as Cn, where 'n' refers to the protonation site as defined in Figure 3 in the main text. In the aminonaphthalene plots, vertical red and black lines are included at wavenumbers 3373 and 1300 cm^{-1}, respectively. The IRMPD spectrum obtained for TIMS-resolved peak C of protonated aminoanthracene is superimposed on all the calculated spectra on page S8 for comparison. The energy of each tautomer in kJ mol^{-1} relative to the lowest-energy C3-protomer is indicated in each panel.*

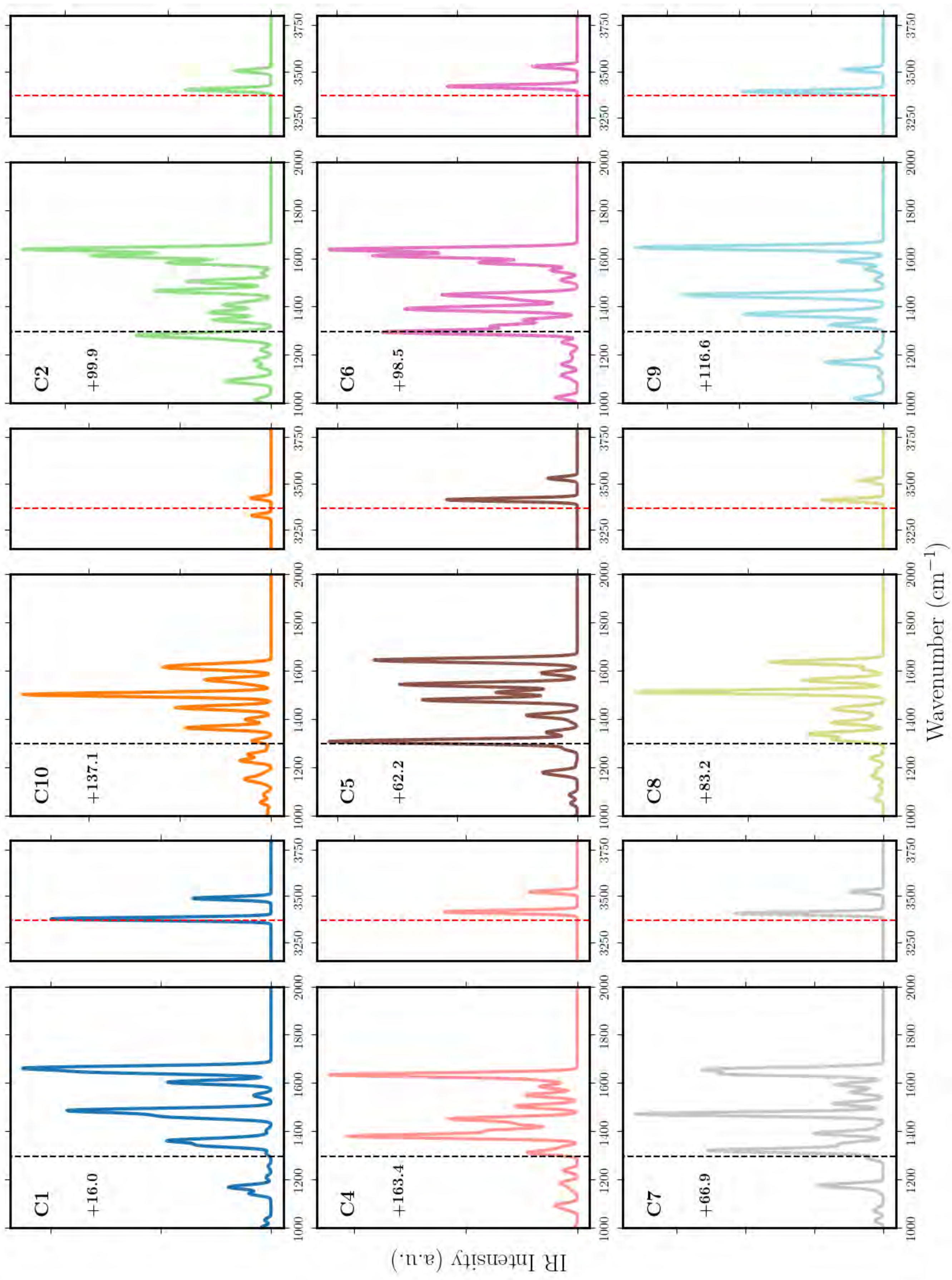
C1
+16.0
C10
+137.1
C2
+99.9
C4
+163.4
C5
+62.2
C6
+98.5
C7
+66.9
C8
+83.2
C9
+116.6
IR Intensity (a.u.)
Wavenumber (cm^{-1})

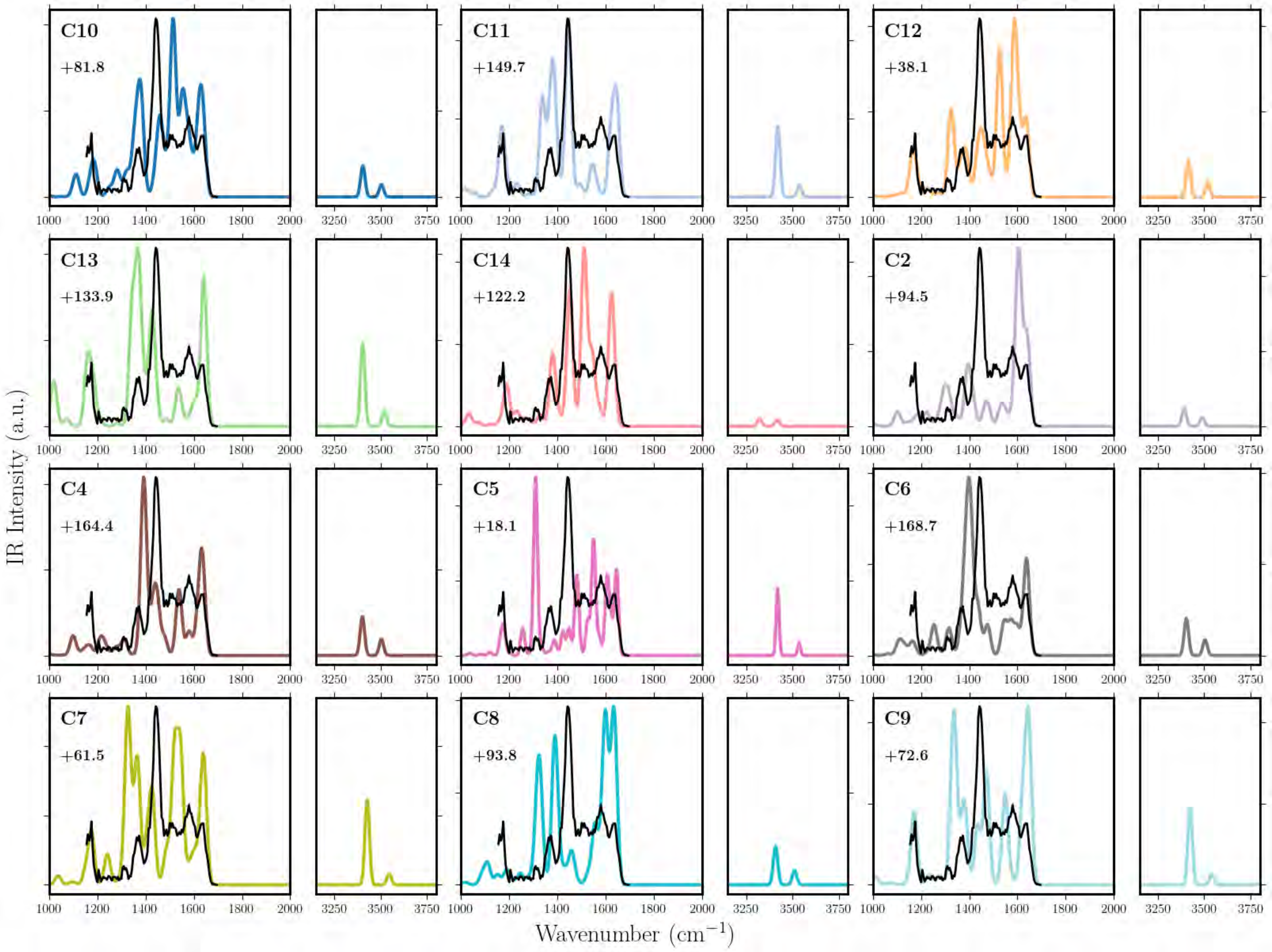
C10
+81.8
C11
+149.7
C12
+38.1
C13
+133.9
C14
+122.2
C2
+94.5
C4
+164.4
C5
+18.1
C6
+168.7
C7
+61.5
C8
+93.8
C9
+72.6
IR Intensity (a.u.)
Wavenumber (cm^{-1})
1000 1200 1400 1600 1800 2000
3250 3500 3750

Supplementary Data 1: Cartesian atomic coordinates of optimized geometries at the B3LYP/6-31++G** level of theory.

1-ANC_3H^+

7	-1.705489000	2.115775000	-0.000009000
6	1.757592000	-1.286200000	-0.000015000
6	2.764419000	-0.326624000	-0.000014000
6	2.441815000	1.040320000	0.000008000
6	1.114620000	1.432503000	0.000020000
6	0.408732000	-0.910924000	-0.000003000
6	-0.656544000	-1.970349000	0.000011000
6	-2.052190000	-1.473496000	0.000000000
6	0.078099000	0.468758000	0.000009000
6	-1.325469000	0.844700000	-0.000001000
6	-2.361281000	-0.159274000	-0.000001000
1	-3.396712000	0.168516000	-0.000009000
1	-0.523697000	-2.637733000	-0.866772000
1	-2.686579000	2.364443000	-0.000021000
1	-1.046803000	2.881779000	-0.000069000
1	3.804709000	-0.636144000	-0.000027000
1	3.228645000	1.786818000	0.000017000
1	2.016909000	-2.340993000	-0.000027000
1	0.896595000	2.495786000	0.000047000
1	-0.523703000	-2.637683000	0.866836000
1	-2.849685000	-2.211696000	-0.000002000

1-ANC_1H^+

7	1.696222000	-2.095524000	0.000002000
6	-1.773896000	1.301085000	-0.000009000
6	-2.777040000	0.339739000	-0.000010000
6	-2.457484000	-1.031654000	0.000002000

6	-1.132931000	-1.424742000	0.000009000
6	-0.418504000	0.931617000	0.000003000
6	0.616643000	1.952605000	0.000013000
6	1.919674000	1.631796000	0.000009000
6	-0.089212000	-0.462534000	0.000007000
6	1.295769000	-0.833290000	0.000002000
6	2.377781000	0.209469000	-0.000016000
1	0.302320000	2.991377000	0.000026000
1	3.025750000	0.031739000	0.872690000
1	3.025660000	0.031766000	-0.872800000
1	1.049091000	-2.872313000	0.000007000
1	2.680619000	-2.332669000	-0.000003000
1	-2.032632000	2.355122000	-0.000015000
1	-3.817808000	0.648372000	-0.000019000
1	-3.246694000	-1.775424000	0.000002000
1	-0.915966000	-2.488471000	0.000018000
1	2.691305000	2.394612000	0.000020000

1-ANNH$_3^+$

7	-1.876756000	-2.012632000	0.000000000
6	1.877708000	1.212762000	0.000000000
6	2.790282000	0.180400000	0.000000000
6	2.345699000	-1.163104000	0.000000000
6	0.997435000	-1.457824000	0.000000000
6	0.480983000	0.952708000	0.000000000
6	-0.475836000	2.004782000	0.000000000
6	-1.829470000	1.748261000	0.000000000
6	0.034478000	-0.412820000	0.000000000
6	-1.371038000	-0.607467000	0.000000000
6	-2.292099000	0.411612000	0.000000000
1	-1.560463000	-2.533588000	0.828247000

1	-1.560476000	-2.533584000	-0.828255000
1	-2.901913000	-2.024773000	0.000008000
1	3.854216000	0.392496000	0.000000000
1	3.072429000	-1.969010000	0.000000000
1	2.215255000	2.244730000	0.000000000
1	0.697531000	-2.503390000	-0.000001000
1	-0.119153000	3.030327000	0.000000000
1	-2.548340000	2.559717000	0.000000000
1	-3.360649000	0.209639000	0.000000000

1-ANC$_5$H$^+$

7	1.949237000	1.994823000	0.000030000
6	0.514365000	-1.952388000	-0.000001000
6	1.896284000	-1.649394000	-0.000018000
6	2.365361000	-0.350398000	-0.000018000
6	1.465079000	0.735909000	0.000001000
6	-1.886961000	-1.228289000	0.000028000
6	-2.797863000	-0.058644000	0.000000000
6	-0.409021000	-0.934860000	0.000010000
6	0.034761000	0.448854000	0.000001000
6	-0.923400000	1.454175000	-0.000023000
6	-2.329412000	1.217584000	-0.000024000
1	-2.143573000	-1.863630000	0.864330000
1	2.946820000	2.151833000	0.000021000
1	1.370035000	2.818511000	0.000074000
1	2.613346000	-2.464835000	-0.000031000
1	0.193638000	-2.989216000	0.000002000
1	-2.143587000	-1.863691000	-0.864224000
1	-3.867044000	-0.253527000	0.000003000
1	-0.616195000	2.496591000	-0.000045000

1	-3.006394000	2.064605000	-0.000045000
1	3.433135000	-0.155702000	-0.000026000

1-ANC$_7$H$^+$

7	1.904682000	-2.024109000	0.000000000
6	0.565728000	1.962550000	-0.000001000
6	1.934385000	1.620361000	-0.000001000
6	2.378139000	0.307373000	0.000000000
6	1.458114000	-0.756600000	0.000001000
6	-1.806107000	1.286221000	0.000002000
6	-2.754903000	0.326500000	0.000002000
6	-0.394739000	0.971185000	0.000000000
6	0.019702000	-0.441771000	0.000001000
6	-0.944033000	-1.408458000	0.000005000
6	-2.394545000	-1.116354000	-0.000004000
1	-2.855038000	-1.633853000	0.861440000
1	-3.809096000	0.585083000	0.000005000
1	2.897663000	-2.211463000	0.000003000
1	1.299799000	-2.829806000	-0.000003000
1	2.673146000	2.416558000	-0.000001000
1	0.270811000	3.006098000	0.000000000
1	-2.083783000	2.335773000	0.000004000
1	-0.683603000	-2.464133000	0.000011000
1	-2.855008000	-1.633828000	-0.861480000
1	3.441876000	0.092289000	-0.000002000

1-ANC$_8$H$^+$

7	-1.772632000	2.095005000	0.043776000
6	-0.650544000	-1.981949000	-0.007503000
6	-1.981688000	-1.610658000	0.008565000
6	-2.334204000	-0.254622000	0.009246000

6	-1.372027000	0.780362000	-0.005974000
6	1.705611000	-1.319077000	-0.008474000
6	2.758932000	-0.373720000	0.007244000
6	0.344746000	-0.964311000	-0.014926000
6	-0.011269000	0.420174000	-0.013647000
6	1.065799000	1.457268000	0.001876000
6	2.459429000	0.960067000	0.014575000
1	0.935317000	2.134518000	0.868047000
1	3.255231000	1.700488000	0.027530000
1	3.788189000	-0.715337000	0.015239000
1	-2.752254000	2.307171000	-0.077014000
1	-1.150141000	2.834326000	-0.242253000
1	-2.764142000	-2.361670000	0.018209000
1	-0.354716000	-3.025589000	-0.010594000
1	1.957635000	-2.377293000	-0.010917000
1	0.971574000	2.141889000	-0.863390000
1	-3.386983000	0.015261000	0.022819000

1-ANC$_6$H$^+$

7	1.861526000	2.071421000	-0.009671000
6	0.625200000	-1.986783000	0.001721000
6	1.955130000	-1.640444000	-0.020360000
6	2.333812000	-0.284636000	-0.014144000
6	1.409049000	0.777887000	0.008349000
6	-1.701914000	-1.273560000	0.023817000
6	-2.770022000	-0.264443000	0.016302000
6	-0.361611000	-0.947453000	0.009337000
6	0.022174000	0.457164000	-0.002545000
6	-1.004319000	1.465392000	-0.035101000
6	-2.323183000	1.148361000	-0.025944000
1	-3.454549000	-0.484552000	-0.825262000

1	2.849305000	2.241533000	0.107618000
1	1.268213000	2.834134000	0.274063000
1	2.726646000	-2.402647000	-0.032981000
1	0.307457000	-3.023874000	0.011648000
1	-1.993858000	-2.321500000	0.040369000
1	-3.428442000	-0.444085000	0.888301000
1	-0.723323000	2.511271000	-0.085527000
1	-3.081619000	1.923819000	-0.055803000
1	3.393597000	-0.042962000	-0.023319000

1-ANC$_2$H$^+$

7	-1.749652000	2.139435000	-0.005042000
6	1.845198000	-1.258988000	0.047841000
6	2.797734000	-0.272219000	0.019195000
6	2.387006000	1.081608000	-0.044824000
6	1.044020000	1.440966000	-0.055660000
6	0.447351000	-0.928416000	0.028934000
6	-0.503114000	-1.930669000	0.008376000
6	-1.930702000	-1.621094000	-0.041546000
6	0.034960000	0.464895000	-0.000938000
6	-1.381325000	0.813589000	0.017218000
6	-2.311710000	-0.197193000	-0.010676000
1	-2.348279000	-2.117875000	-0.941350000
1	-0.197290000	-2.973787000	0.014504000
1	-3.371856000	0.037344000	-0.013264000
1	-1.153406000	2.827120000	0.430283000
1	-2.734067000	2.348092000	0.086662000
1	2.128652000	-2.306736000	0.077912000
1	3.853825000	-0.518279000	0.031151000
1	3.141064000	1.861681000	-0.092708000
1	0.792543000	2.491886000	-0.132160000

1	-2.440131000	-2.180373000	0.766748000

1-ANC$_9$H$^+$

7	-1.817846000	2.031438000	-0.106586000
6	-0.546138000	-1.971668000	-0.001484000
6	-1.907252000	-1.619229000	-0.044377000
6	-2.349581000	-0.299709000	-0.065589000
6	-1.431339000	0.757262000	-0.005507000
6	1.823422000	-1.275092000	-0.022445000
6	2.749152000	-0.270684000	-0.109446000
6	0.428528000	-0.990163000	0.064370000
6	0.010007000	0.442701000	0.308414000
6	1.064390000	1.477803000	0.055563000
6	2.359236000	1.116922000	-0.099100000
1	-0.069255000	0.437917000	1.426149000
1	0.806929000	2.530523000	0.107072000
1	3.122675000	1.878382000	-0.221115000
1	3.801258000	-0.516339000	-0.213447000
1	-2.798281000	2.267106000	-0.191691000
1	-1.163611000	2.796389000	-0.173617000
1	-2.648378000	-2.410151000	-0.114017000
1	-0.257077000	-3.015015000	-0.070459000
1	2.132298000	-2.314314000	-0.079712000
1	-3.404188000	-0.083416000	-0.205462000

1-ANC$_4$H$^+$

7	-1.868990000	2.056556000	0.028099000
6	-0.573397000	-2.000221000	-0.015571000
6	-1.888449000	-1.657442000	-0.146594000
6	-2.300949000	-0.304480000	-0.113154000
6	-1.411244000	0.767696000	0.030397000

6	1.875128000	-1.216368000	0.018041000
6	2.775332000	-0.202872000	-0.095925000
6	0.430562000	-0.936579000	0.222690000
6	-0.005785000	0.481525000	0.064127000
6	0.987633000	1.478795000	-0.028936000
6	2.328710000	1.152254000	-0.111801000
1	-2.864708000	2.221508000	0.041784000
1	-1.309344000	2.807558000	0.399621000
1	-2.640617000	-2.429085000	-0.273912000
1	-0.248906000	-3.034805000	0.025350000
1	2.193935000	-2.254367000	0.041701000
1	3.833176000	-0.419629000	-0.200475000
1	0.705424000	2.522555000	-0.106246000
1	3.061443000	1.946815000	-0.213757000
1	0.409057000	-1.043917000	1.352510000
1	-3.361773000	-0.086378000	-0.202915000

1-AAC$_3$H$^+$

7	-2.565270000	2.289429000	0.000003000
6	2.854510000	-1.334441000	0.000001000
6	3.965257000	-0.518874000	0.000001000
6	3.831638000	0.896780000	-0.000001000
6	2.585820000	1.475763000	-0.000001000
6	0.381035000	-1.570889000	0.000001000
6	1.551182000	-0.771624000	0.000001000
6	1.413855000	0.661157000	-0.000001000
6	0.122710000	1.213320000	-0.000001000
6	-0.883421000	-1.021806000	0.000000000

6	-2.095004000	-1.917869000	-0.000001000
6	-3.402838000	-1.218878000	-0.000001000
6	-1.022783000	0.409992000	0.000000000
6	-2.356095000	0.977043000	0.000001000
6	-3.522094000	0.125466000	0.000000000
1	-2.068569000	-2.595483000	0.867970000
1	2.964678000	-2.414657000	0.000002000
1	4.957708000	-0.958509000	0.000002000
1	0.052459000	2.297545000	-0.000003000
1	-1.811908000	2.961833000	0.000003000
1	-3.505055000	2.663836000	-0.000002000
1	4.721738000	1.517329000	-0.000001000
1	2.478894000	2.556756000	-0.000003000
1	0.493837000	-2.652266000	0.000001000
1	-2.068568000	-2.595481000	-0.867974000
1	-4.299593000	-1.833038000	-0.000002000
1	-4.501367000	0.595294000	0.000000000

1-AAC_1H^+

7	-2.570788000	2.257940000	-0.000069000
6	2.869283000	-1.332906000	-0.000019000
6	3.976526000	-0.510416000	-0.000003000
6	3.838760000	0.904840000	0.000021000
6	2.590204000	1.477831000	0.000028000
6	0.399458000	-1.584850000	-0.000022000
6	1.563894000	-0.778451000	-0.000012000
6	1.421826000	0.656998000	0.000011000
6	0.131860000	1.204229000	0.000015000
6	-0.873367000	-1.044160000	-0.000012000
6	-2.048360000	-1.903050000	-0.000016000

6	-3.292897000	-1.404326000	0.000018000
6	-1.017543000	0.397624000	0.000002000
6	-2.337871000	0.952728000	-0.000007000
6	-3.555617000	0.069212000	0.000063000
1	-4.170130000	0.338789000	0.873491000
1	2.985947000	-2.412279000	-0.000037000
1	4.970750000	-0.946181000	-0.000010000
1	0.058510000	2.288572000	0.000041000
1	-1.830287000	2.945654000	-0.000122000
1	-3.516900000	2.617701000	-0.000070000
1	4.726559000	1.528465000	0.000033000
1	2.477761000	2.558254000	0.000046000
1	0.514561000	-2.665034000	-0.000036000
1	-1.885930000	-2.976378000	-0.000043000
1	-4.161970000	-2.053786000	0.000025000
1	-4.170283000	0.338828000	-0.873243000

1-AAC$_5$H$^+$

7	2.622448000	2.291405000	0.065163000
6	-2.893655000	-1.266356000	0.018902000
6	-3.978180000	-0.385868000	-0.032864000
6	-3.781973000	1.005444000	-0.066312000
6	-2.493645000	1.509678000	-0.049298000
6	-0.407146000	-1.706698000	0.109098000
6	-1.588117000	-0.778764000	0.036706000
6	-1.383221000	0.627286000	0.002773000
6	-0.058041000	1.140187000	0.013050000
6	0.962634000	-1.079561000	0.025795000
6	2.086724000	-1.868195000	-0.043159000
6	3.368306000	-1.271853000	-0.094130000

6	1.094649000	0.363096000	0.033647000
6	2.425449000	0.956075000	0.015355000
6	3.542379000	0.097719000	-0.064269000
1	-0.499504000	-2.474148000	-0.670685000
1	-3.071875000	-2.337682000	0.042914000
1	-4.987659000	-0.785202000	-0.049113000
1	0.022500000	2.224255000	-0.019532000
1	1.881286000	2.960550000	0.192815000
1	3.560848000	2.663255000	0.045905000
1	-4.634281000	1.674962000	-0.106492000
1	-2.321358000	2.581969000	-0.076156000
1	-0.468039000	-2.264048000	1.056918000
1	1.999239000	-2.949859000	-0.059845000
1	4.244433000	-1.910743000	-0.152567000
1	4.540305000	0.523714000	-0.092070000

1-AAC$_{12}$H^{+}

7	2.694268000	2.235054000	-0.046175000
6	-2.837073000	-1.341811000	0.003386000
6	-3.968042000	-0.549360000	-0.009408000
6	-3.828836000	0.851475000	-0.017011000
6	-2.568933000	1.456386000	-0.011010000
6	-0.384731000	-1.542515000	0.017297000
6	-1.545880000	-0.742860000	0.008804000
6	-1.412586000	0.678763000	0.001738000
6	-0.048727000	1.311580000	0.010311000
6	0.927446000	-1.032962000	0.016895000
6	2.027540000	-1.935023000	0.002602000
6	3.312056000	-1.423460000	-0.018851000
6	1.125741000	0.378901000	0.016598000

6	2.440632000	0.882029000	0.004118000
6	3.512880000	-0.038356000	-0.017813000
1	0.007526000	1.982058000	0.885112000
1	1.994544000	2.893575000	0.258684000
1	3.644023000	2.549699000	0.088811000
1	-4.955659000	-0.997442000	-0.014086000
1	-2.918221000	-2.424812000	0.008700000
1	-4.717040000	1.475997000	-0.028108000
1	-2.494347000	2.540056000	-0.017522000
1	-0.509639000	-2.623809000	0.019376000
1	0.033884000	1.985347000	-0.859221000
1	1.846615000	-3.004726000	0.004793000
1	4.170752000	-2.085804000	-0.033736000
1	4.528776000	0.347768000	-0.035501000

1-AANH$^+$

7	-2.626331000	2.268902000	0.000001000
6	2.892473000	-1.306586000	0.000000000
6	3.976656000	-0.467257000	0.000000000
6	3.795792000	0.947658000	0.000000000
6	2.537058000	1.494178000	0.000000000
6	0.435585000	-1.610013000	0.000000000
6	1.563456000	-0.778951000	0.000000000
6	1.381300000	0.655467000	0.000000000
6	0.077533000	1.179772000	0.000000000
6	-0.866896000	-1.094031000	0.000000000
6	-2.014643000	-1.945244000	0.000000000
6	-3.290286000	-1.438259000	0.000001000
6	-1.045933000	0.342963000	-0.000001000
6	-2.399203000	0.795151000	-0.000001000

6	-3.490350000	-0.030482000	-0.000001000
1	-2.211976000	2.717478000	0.827803000
1	-2.211975000	2.717479000	-0.827799000
1	-3.629369000	2.479738000	0.000000000
1	4.982179000	-0.875364000	0.000001000
1	3.026492000	-2.384258000	0.000000000
1	4.667413000	1.594550000	0.000000000
1	2.406495000	2.572826000	-0.000001000
1	0.572224000	-2.688077000	0.000000000
1	-0.020174000	2.264100000	0.000001000
1	-1.858584000	-3.019884000	0.000000000
1	-4.151714000	-2.096093000	0.000002000
1	-4.501945000	0.368988000	0.000000000

1-AAC$_7$H$^+$

7	2.723523000	2.245510000	0.000005000
6	-2.911026000	-1.327082000	0.000008000
6	-3.998760000	-0.311224000	-0.000011000
6	-3.758666000	1.029829000	-0.000008000
6	-2.418201000	1.509768000	0.000005000
6	-0.400246000	-1.583795000	-0.000004000
6	-1.504036000	-0.777740000	0.000002000
6	-1.313683000	0.657841000	0.000008000
6	0.001683000	1.192863000	0.000009000
6	0.932756000	-1.063152000	-0.000003000
6	2.042607000	-1.909613000	-0.000004000
6	3.331003000	-1.367144000	0.000000000
6	1.124811000	0.381499000	0.000001000
6	2.484606000	0.911006000	-0.000001000
6	3.556005000	0.008483000	0.000001000

1	-3.064819000	-1.991394000	0.866337000
1	-5.021399000	-0.679747000	-0.000022000
1	0.086816000	2.275654000	0.000020000
1	1.995612000	2.939629000	-0.000061000
1	3.672180000	2.589019000	-0.000026000
1	-3.064815000	-1.991440000	-0.866284000
1	-4.577897000	1.740601000	-0.000017000
1	-2.250291000	2.584283000	0.000009000
1	-0.515697000	-2.664800000	-0.000008000
1	1.902443000	-2.985137000	-0.000006000
1	4.187681000	-2.034230000	-0.000001000
1	4.572397000	0.389753000	0.000001000

1-AAC$_9$H$^+$

7	2.712968000	2.255772000	0.000006000
6	-2.852054000	-1.342038000	0.000000000
6	-3.930390000	-0.532999000	0.000005000
6	-3.804149000	0.956810000	0.000005000
6	-2.415090000	1.467468000	-0.000003000
6	-0.381420000	-1.608774000	-0.000004000
6	-1.500397000	-0.813208000	-0.000003000
6	-1.321996000	0.637360000	-0.000004000
6	0.004170000	1.181228000	-0.000005000
6	0.942049000	-1.074090000	-0.000002000
6	2.061383000	-1.905046000	0.000001000
6	3.345164000	-1.348909000	0.000005000
6	1.124342000	0.380794000	-0.000003000
6	2.485691000	0.922202000	-0.000003000
6	3.563074000	0.028027000	0.000002000
1	-4.340212000	1.394590000	-0.861826000

1	0.080547000	2.264910000	-0.000009000
1	1.980022000	2.945025000	-0.000010000
1	3.659261000	2.606894000	0.000008000
1	-4.933143000	-0.948723000	0.000009000
1	-2.973748000	-2.421174000	-0.000001000
1	-4.340199000	1.394588000	0.861845000
1	-2.272740000	2.546487000	-0.000006000
1	-0.490688000	-2.689472000	-0.000006000
1	1.933945000	-2.981998000	0.000000000
1	4.206873000	-2.009703000	0.000003000
1	4.577056000	0.415223000	0.000003000

1-AAC$_{10}$H$^+$

7	-2.631280000	2.298391000	-0.005743000
6	2.747206000	-1.357277000	-0.040057000
6	3.938331000	-0.585750000	-0.017745000
6	3.858199000	0.776560000	0.028588000
6	2.566931000	1.509179000	0.054166000
6	0.319997000	-1.605314000	-0.032396000
6	1.469910000	-0.783280000	-0.025577000
6	1.325318000	0.654775000	0.012626000
6	0.060863000	1.189289000	0.020824000
6	-0.966584000	-1.063606000	-0.012790000
6	-2.114537000	-1.911659000	0.014738000
6	-3.366601000	-1.344108000	0.050242000
6	-1.110065000	0.376607000	-0.007906000
6	-2.428615000	0.931741000	-0.016762000
6	-3.519930000	0.057211000	0.029156000
1	2.566211000	2.154334000	0.949585000
1	-0.044405000	2.268449000	0.077065000

1	-1.957337000	2.896199000	-0.459882000
1	-3.582445000	2.617714000	-0.124973000
1	4.900310000	-1.086291000	-0.033245000
1	2.828214000	-2.441700000	-0.067217000
1	4.767341000	1.372440000	0.051225000
1	2.573682000	2.231306000	-0.779578000
1	0.443141000	-2.685071000	-0.046497000
1	-1.982452000	-2.988425000	0.011751000
1	-4.252905000	-1.969084000	0.079819000
1	-4.522943000	0.475178000	0.039498000

1-AAC_8H^+

7	2.654416000	2.291382000	0.006640000
6	-2.734958000	-1.323357000	0.038083000
6	-3.961225000	-0.504089000	0.015715000
6	-3.747952000	0.972056000	-0.032849000
6	-2.509207000	1.509490000	-0.049267000
6	-0.314761000	-1.598066000	0.029350000
6	-1.475211000	-0.768294000	0.022825000
6	-1.320656000	0.683652000	-0.016389000
6	-0.042103000	1.205808000	-0.024869000
6	0.965308000	-1.062546000	0.010659000
6	2.111821000	-1.918265000	-0.013708000
6	3.364396000	-1.357319000	-0.045814000
6	1.115729000	0.384966000	0.004834000
6	2.440847000	0.930052000	0.016611000
6	3.524000000	0.045801000	-0.025350000
1	-4.583023000	-0.785334000	0.886471000
1	0.064993000	2.283818000	-0.080075000
1	1.969214000	2.904819000	0.420293000

1	3.604531000	2.611457000	0.129339000
1	-4.587529000	-0.840612000	-0.831713000
1	-2.845598000	-2.405798000	0.065371000
1	-4.628850000	1.605508000	-0.055223000
1	-2.379386000	2.587154000	-0.085821000
1	-0.443207000	-2.677370000	0.042849000
1	1.972940000	-2.994084000	-0.010277000
1	4.248737000	-1.985049000	-0.072515000
1	4.530085000	0.456477000	-0.034165000

1-AAC$_2$H$^+$

7	2.560682000	2.335306000	-0.017360000
6	-2.900163000	-1.322154000	-0.052629000
6	-3.987513000	-0.488582000	-0.012486000
6	-3.801660000	0.922168000	0.049379000
6	-2.539529000	1.482202000	0.067078000
6	-0.446956000	-1.588197000	-0.052162000
6	-1.573417000	-0.778544000	-0.035207000
6	-1.388715000	0.658755000	0.021134000
6	-0.078121000	1.204664000	0.031343000
6	0.869278000	-1.039865000	-0.029891000
6	1.960265000	-1.882789000	0.014560000
6	3.328678000	-1.362418000	0.077189000
6	1.051988000	0.410689000	-0.012107000
6	2.410452000	0.960256000	-0.027225000
6	3.479650000	0.110602000	0.019441000
1	3.922244000	-1.853686000	-0.716662000
1	-3.024898000	-2.399942000	-0.096487000
1	-4.993095000	-0.895147000	-0.025335000
1	0.009443000	2.283214000	0.101155000

1	1.900469000	2.887708000	-0.546291000
1	3.507048000	2.681562000	-0.100711000
1	-4.673709000	1.568054000	0.083586000
1	-2.420248000	2.560140000	0.114884000
1	-0.558396000	-2.669269000	-0.073046000
1	1.814654000	-2.960417000	0.019695000
1	3.795759000	-1.776651000	0.993592000
1	4.490538000	0.506566000	0.016626000

1-AA$C_{13}H^+$

7	2.591738000	2.264451000	-0.153184000
6	-2.847625000	-1.330697000	-0.083036000
6	-3.940785000	-0.504829000	-0.134617000
6	-3.798292000	0.922525000	-0.088902000
6	-2.563445000	1.491084000	0.012601000
6	-0.399575000	-1.611224000	0.033983000
6	-1.530287000	-0.789834000	0.015618000
6	-1.378529000	0.666541000	0.071239000
6	-0.126268000	1.217887000	0.167640000
6	0.903573000	-1.104399000	0.095436000
6	2.046369000	-1.914528000	0.014452000
6	3.309626000	-1.340842000	-0.080357000
6	1.088565000	0.377610000	0.330173000
6	2.443385000	0.931081000	-0.050334000
6	3.512768000	0.049531000	-0.143390000
1	1.217715000	0.399405000	1.443288000
1	-2.968818000	-2.408809000	-0.119923000
1	-4.935823000	-0.931719000	-0.211521000
1	-0.041765000	2.297279000	0.249768000
1	1.809669000	2.885333000	-0.290878000

1	3.510014000	2.663393000	-0.294071000
1	-4.685973000	1.544940000	-0.130980000
1	-2.448955000	2.570087000	0.054829000
1	-0.530783000	-2.686431000	-0.051533000
1	1.932356000	-2.992568000	-0.028176000
1	4.176531000	-1.988760000	-0.167249000
1	4.506792000	0.437251000	-0.344309000

1-AA$C_{11}H^+$

7	-2.695439000	2.248629000	-0.087832000
6	2.868456000	-1.327419000	-0.006543000
6	3.928509000	-0.481740000	-0.168005000
6	3.751207000	0.953870000	-0.230994000
6	2.538424000	1.514890000	-0.036199000
6	0.410264000	-1.610887000	0.040940000
6	1.532961000	-0.817932000	0.111583000
6	1.367853000	0.655740000	0.356163000
6	-0.003306000	1.185661000	0.161616000
6	-0.909441000	-1.081600000	0.029169000
6	-2.027507000	-1.915102000	-0.020775000
6	-3.309035000	-1.362277000	-0.069607000
6	-1.099206000	0.380877000	0.044921000
6	-2.466199000	0.916253000	-0.053126000
6	-3.534191000	0.016624000	-0.088734000
1	1.419752000	0.730228000	1.478202000
1	-0.095995000	2.267512000	0.213082000
1	-1.964322000	2.935499000	-0.168110000
1	-3.641052000	2.593937000	-0.164247000
1	4.926641000	-0.888458000	-0.295764000
1	3.011556000	-2.403397000	-0.036410000

1	4.609481000	1.579072000	-0.455308000
1	2.399133000	2.591775000	-0.063607000
1	0.525209000	-2.687485000	-0.049415000
1	-1.896998000	-2.991608000	-0.023083000
1	-4.168280000	-2.025449000	-0.104432000
1	-4.549793000	0.396221000	-0.138541000

1-AAC_4H^+

7	-2.617741000	2.301961000	0.062227000
6	2.896641000	-1.302610000	0.016450000
6	3.969473000	-0.459829000	-0.087541000
6	3.769519000	0.952402000	-0.141151000
6	2.505860000	1.501757000	-0.094544000
6	0.458814000	-1.595777000	0.127411000
6	1.560189000	-0.771754000	0.067814000
6	1.362413000	0.669555000	0.007852000
6	0.053587000	1.204042000	0.025306000
6	-0.912505000	-1.071938000	0.265575000
6	-2.078343000	-1.930190000	-0.092169000
6	-3.293999000	-1.350334000	-0.269337000
6	-1.078236000	0.409773000	0.101122000
6	-2.423945000	0.940386000	0.036962000
6	-3.476059000	0.061229000	-0.174989000
1	-0.970272000	-1.204527000	1.389255000
1	3.034083000	-2.378635000	0.062719000
1	4.978182000	-0.857101000	-0.125840000
1	-0.046732000	2.278208000	-0.090072000
1	-1.992118000	2.878528000	0.605318000
1	-3.570983000	2.636831000	0.051126000
1	4.634058000	1.604145000	-0.221197000

1	2.375071000	2.578498000	-0.138890000
1	0.587754000	-2.675171000	0.159599000
1	-1.945089000	-3.006815000	-0.099487000
1	-4.160316000	-1.969976000	-0.478162000
1	-4.479897000	0.462012000	-0.282505000

1-AAC$_6$H$^+$

7	2.627145000	2.293086000	0.027756000
6	-2.885990000	-1.318455000	-0.006048000
6	-3.933309000	-0.479210000	-0.188003000
6	-3.749302000	0.950098000	-0.180667000
6	-2.506626000	1.513555000	-0.050313000
6	-0.327462000	-1.606302000	0.116622000
6	-1.528673000	-0.769942000	0.299086000
6	-1.342460000	0.700554000	0.084161000
6	-0.051415000	1.209633000	0.039527000
6	0.938816000	-1.068192000	0.047880000
6	2.085567000	-1.920600000	-0.070494000
6	3.329787000	-1.355588000	-0.181259000
6	1.093640000	0.386384000	0.049568000
6	2.423540000	0.930033000	0.006499000
6	3.496373000	0.048995000	-0.130860000
1	-1.538846000	-0.845913000	1.428295000
1	-3.016465000	-2.396474000	0.007535000
1	-4.926399000	-0.881534000	-0.359957000
1	0.062461000	2.281994000	-0.081226000
1	1.984615000	2.878525000	0.540312000
1	3.583822000	2.614436000	0.077161000
1	-4.617195000	1.588647000	-0.310874000
1	-2.384760000	2.591322000	-0.102680000

1	-0.453947000	-2.686347000	0.132216000
1	1.949950000	-2.996737000	-0.069756000
1	4.209983000	-1.982070000	-0.280158000
1	4.501846000	0.456761000	-0.189354000
